# Patient-Centered Televisit for Chronic Obstructive Pulmonary Disease Discharge Transitions: User-Centered Design Study

**DOI:** 10.2196/77953

**Published:** 2025-12-05

**Authors:** Joanna Abraham, Alicia Meng, Nicolas Caravelli, Leah Traeger, May Nguyen, Vineet Arora, Valerie G Press

**Affiliations:** 1 Department of Anesthesiology, School of Medicine, Washington University in St. Louis, 660 S Euclid Ave, St. Louis, MO, 63110, United States, 1 3143625129; 2 Department of Medicine, University of Chicago, Chicago, IL, United States

**Keywords:** virtual, remote, chronic obstructive pulmonary disease, readmission, telehealth, telemedicine, participatory design, education

## Abstract

**Background:**

Chronic obstructive pulmonary disease (COPD) affects approximately 16 million Americans and often results in avoidable readmissions due, in part, to medication errors and lack of education. Telehealth interventions can support medication reconciliation and inhaler education following hospital discharge for patients with COPD.

**Objective:**

This study aimed to design and prototype TELE-TOC (Telehealth Education: Leveraging Electronic Transitions of Care), a post-discharge, in-home, televisit intervention, and to map its workflow to ensure integration into the routine discharge care transition process for patients with COPD.

**Methods:**

A user-centered design approach across 3 phases was followed to develop and prototype TELE-TOC. Participants included adult patients hospitalized for COPD exacerbations, their caregivers, clinicians involved in COPD care, and organizational leaders. Data collection methods included semi-structured interviews, system usability scale surveys, and cognitive walkthroughs of the TELE-TOC prototype to assess participants’ perceptions on usability and feasibility of TELE-TOC implementation as part of routine COPD discharge care transitions. Qualitative data were analyzed using inductive thematic analysis and an inductive-deductive approach guided by the Agency for Healthcare Research and Quality–endorsed Care Transitions Framework. Quantitative data were summarized using basic descriptive statistics.

**Results:**

Participants included 18 patients, 18 clinicians, 8 organizational leaders, and 2 caregivers. Phase 1 identified 3 interdependent stages of COPD hospital-to-home discharge: inpatient pre-discharge, at-home post-discharge, and outpatient clinic visit post-discharge. Key facilitators of discharge care transitions included the hospital’s “meds-to-beds” program and high patient health literacy, while barriers to discharge included poor timing of education and conflicting patient priorities. Phase 2 delineated the core televisit components (eg, dedicated clinician, medication reconciliation, inhaler use, and self-management education) and flexible components (eg, reminder system and session frequency). Potential implementation enablers included multiple techniques for clinicians to access and support patient education and backup communication strategies in the event of technical issues. Potential implementation barriers included insufficient patient technology access and limited technology and health literacy, as well as limited clinician bandwidth for thorough COPD education and medication reconciliation. Phase 3 TELE-TOC prototype walkthroughs demonstrated a positive patient experience (average system usability scale score of 97.5/100), attributed to the benefits of videoconferencing technology for hands-on teaching and the use of the virtual teach-back method. Identified barriers included varying levels of patient technology literacy, insufficient inhaler education, limited patient understanding of medication lists, and clinician uncertainty around TELE-TOC documentation. Suggestions for mitigating these barriers included patient training for TELE-TOC sessions, amendments to pharmacists’ “visit note,” and enhanced patient preparation for medication reconciliation.

**Conclusions:**

Using a co-design approach, we integrated multiple perspectives to develop and optimize TELE-TOC, a patient-centered televisit intervention aimed at supporting discharge care transitions to improve continuity of care and outcomes for patients with COPD. Future research will evaluate the impact of TELE-TOC on readmissions from acute exacerbations.

## Introduction

Chronic obstructive pulmonary disease (COPD) affects over 16 million Americans [1]. A leading cause of hospital readmissions [2] and mortality [3], COPD is projected to cost American health care systems over US $60 billion by 2029 [4]. To reduce costs related to preventable readmissions after an index admission for COPD [5], the Centers for Medicare and Medicaid Services included COPD as part of the Hospital Readmissions Reduction Program (HRRP) financial penalty to reduce hospital readmissions and their associated costs [6,7].

Several factors contribute to readmissions, including inadequate patient self-management of COPD and suboptimal care following a hospital discharge. These factors are often attributed to poor implementation of COPD care guidelines among clinicians during discharge [8], fragmented and inadequate discharge education on strategies to improve skills for COPD self-management at home [9,10], lack of follow-up with patients [11], and insufficient understanding about proper use of medications, including inhaler techniques [12-14]. To address these gaps in medication and symptom self-management, care transition interventions have been explored by many, including our team [15,16]. For instance, pharmacist-led medication reconciliation programs at discharge have been shown to reduce medication discrepancies and 30-day readmission rates for older patients with COPD [17]. Similarly, inhaler technique education using a “Teach-To-Goal” (TTG) approach, consisting of rounds of assessment and education prior to discharge, was shown to reduce all-cause readmissions after initial admissions for COPD compared to simply providing verbal instruction [13,16].

While promising, both medication reconciliation and TTG at discharge can be resource-intensive and challenging to implement at large scale within existing hospital workflows [16,17]. Moreover, although in-hospital pre-discharge interventions may improve patient self-management and potentially reduce readmissions, medication errors still occur in more than half of patients after hospital discharge [18]. Inpatient-only care is likely insufficient for many patients, and researchers recommend that interventions (eg, medication reconciliation and education) extend post-discharge for sustaining medication and COPD self-management skills [19].

To address sustainability and effectiveness concerns, our team helped to develop a pharmacist-led, at-home telehealth program, which has improved inhaler technique and quality of life among patients with COPD, while reducing symptoms [20,21]. Other research has also shown that post-discharge televisit medication reconciliation reduces medication errors across populations [22,23]. The virtual approach for medication review and reconciliation and teaching inhaler technique could enhance COPD education, medication management, and care quality without increasing costs. Informed by published evidence [20,21,24], we developed an end user–informed, comprehensive, post-discharge televisit service termed TELE-TOC (Telehealth Education: Leveraging Electronic Transitions of Care) to facilitate inhaler education and reinforcement of COPD self-management skills and provide opportunities for medication reconciliation and counseling by clinical pharmacists. Here we report on the development and prototyping of the TELE-TOC intervention and identify the components and implementation process necessary to fit TELE-TOC into our current COPD HRRP and DTOC (Discharge Transitions of Care) workflow.

## Methods

### Study Setting

The study was conducted at the University of Chicago Medicine (UCM). We are developing the TELE-TOC intervention for integration into our existing COPD HRRP [25].

### Study Approach

**Figure 1.**
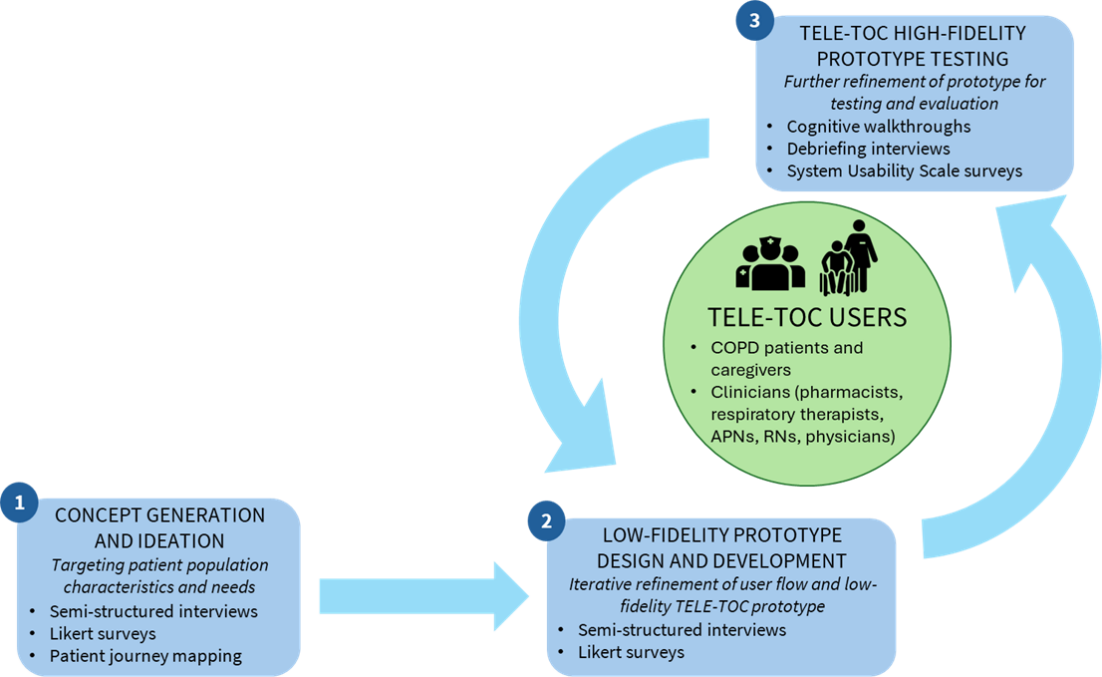
User-centered design phases for Telehealth Education: Leveraging Electronic Transitions Of Care development. APN: advanced practice nurse; COPD: chronic obstructive pulmonary disease; RN: registered nurse; TELE-TOC: Telehealth Education: Leveraging Electronic Transitions of Care.

We followed a user-centered design (UCD) approach to ensure TELE-TOC met the needs, preferences, and behaviors of end users (eg, patients, clinicians, and organizational leaders [OLs]) while considering usability and accessibility (eg, older age, limited health, and technology literacy) [26,27]. Multiple end users were actively engaged in the development of TELE-TOC across 3 iterative phases (Figure 1). Phase 1 mapped the current hospital-home-outpatient care transition workflow for patients with COPD, with emphasis on perceived workflow facilitators and barriers from multiple perspectives (ie, patient, caregiver, clinician, and OLs). Data-informed strategies were then developed to address care transition barriers and inform the TELE-TOC role within the current workflow. Phase 2 identified design ideas and implementation considerations using a low-fidelity TELE-TOC prototype with emphasis on potential facilitators and barriers to adoption and implementation. Phase 3 focused on evaluating the usability and feasibility of a high-fidelity TELE-TOC prototype.

### Participants

Phases 1 and 2: (1) adult patients admitted for COPD exacerbations enrolled in our COPD HRRP, (2) clinicians (pharmacists, advanced practice nurses [APNs], hospitalists, primary care physicians, pulmonologists, and respiratory therapists), and (3) OLs (patient safety and quality improvement hospital leaders). Phase 1 also included patient caregivers. Phase 3: TELE-TOC end users (adult patients and inpatient clinical pharmacists). While we planned to include caregivers in all phases, they were ultimately only included in Phase 1 due to challenges with further caregiver participation (eg, patient discomfort with caregiver involvement, lack of caregiver availability, passive caregiver, and lack of caregiver altogether).

### Data Collection

We used semi-structured interviews to gather participant perspectives on current discharge care transitions. Interview guides were iteratively developed and refined with input from pilot interviews with 2 patients and caregivers from the COPD Foundation, a clinician involved in caring for patients with COPD, and an OL.

**Figure 2.**
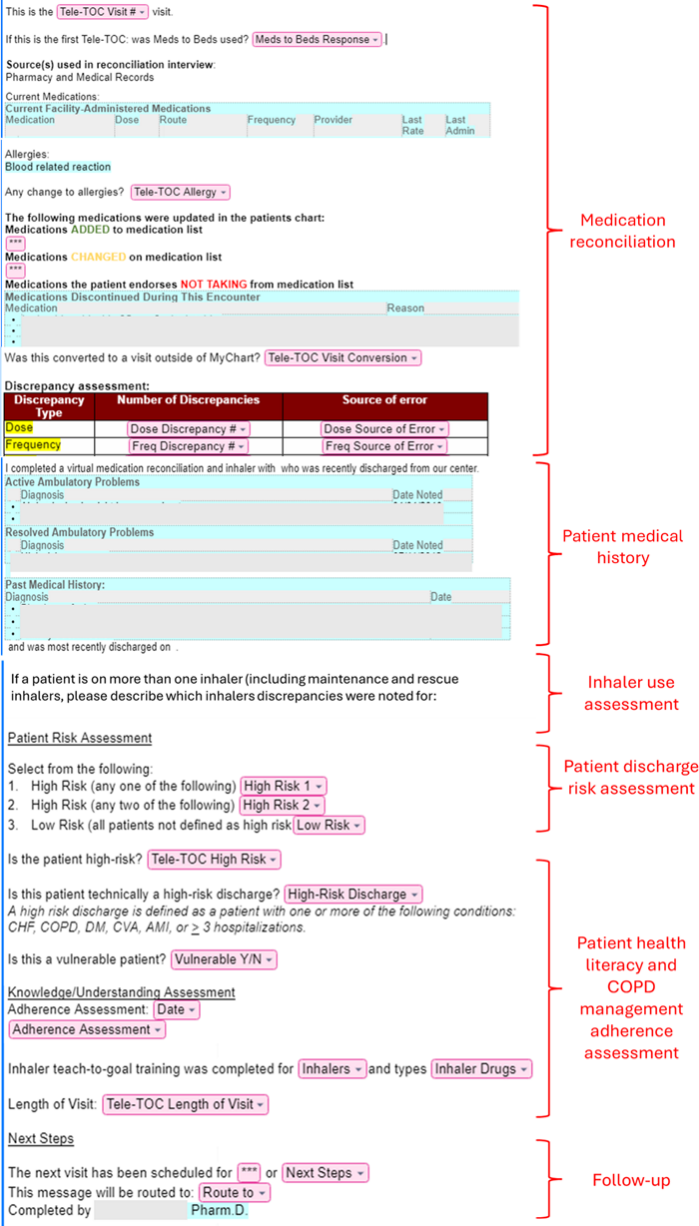
Screenshot of electronic health record–embedded “visit note.” COPD: chronic obstructive pulmonary disease.

Phase 1 mapped current DTOC workflows, ascertained facilitators and barriers, and delineated potential for televisit interventions. Phase 2 assessed attitudes toward the proposed televisit intervention, a low-fidelity prototype of PowerPoint slides with process maps and instructions for inhaler education and identified potential facilitators and barriers to implementation. We asked participants to rate their experiences with medication reconciliation and COPD medication education using a Likert scale from 1 to 5, where 1 indicated “not prepared” or “not good” and 5 indicated “prepared” or “very good.” We requested patient feedback on preferred televisit format (phone, text, or video), comfort levels with video technology, experiences with technical difficulties, and confidence in internet usability. Phase 3 comprised televisit walkthrough usability sessions with 4 patients post-discharge from UCM and 2 clinical pharmacists using the TELE-TOC prototype, as previous research demonstrated that only 3‐5 sessions were required to identify most usability issues [28]. High-fidelity TELE-TOC prototypes consisted of interactive web-based sessions integrated into MyChart (Epic Systems). Additionally, pharmacists piloted a TELE-TOC–specific templated “visit note” in the electronic health record (EHR) to document medication reconciliation and inhaler education (Figure 2). Participants verbalized their actions during the TELE-TOC process. After medication review and inhaler education, patients rated TELE-TOC usability on the system usability scale (SUS) [29] and completed debriefing interviews to assess user satisfaction and suggestions for improvement. Walkthroughs were conducted and recorded via a HIPAA (Health Insurance Portability and Accountability Act)–compliant Zoom platform (Zoom Video Communications Inc) (refer to guides in Multimedia Appendix 1).

### Data Coding and Analysis

We followed an inductive (data-driven) thematic analysis approach for Phase 1 [30]. Two researchers (JA and EZ) independently read the data transcripts multiple times and openly coded the data with keywords representing the data, then identified relationships among codes, highlighting areas of similarity and overlap to form subthemes; repeated patterns among established subthemes identified overarching themes. Additionally, interview responses informed the development of a process map for care transitions of patients with COPD.

**Figure 3.**
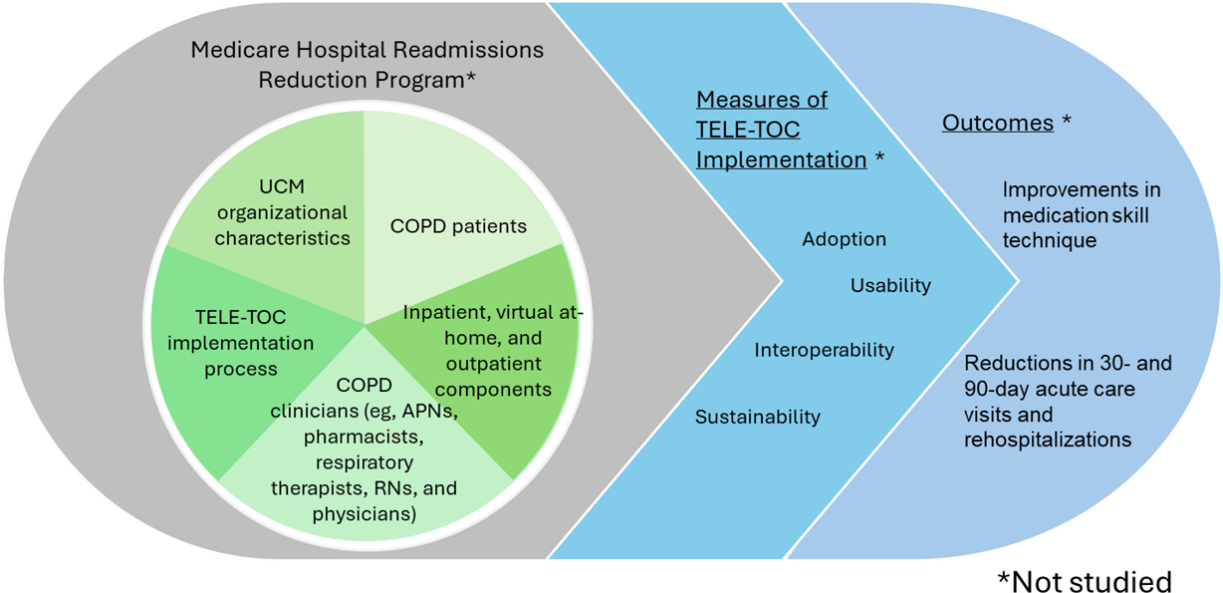
Domains of the Agency for Healthcare Research and Quality–endorsed care transitions framework. APN: advanced practice nurse; COPD: chronic obstructive pulmonary disease; RN: registered nurse; TELE-TOC: Telehealth Education: Leveraging Electronic Transitions of Care; UCM: University of Chicago Medicine.

In Phase 2, we used an inductive-deductive thematic analysis approach to integrate insights on current discharge care transition processes with elements of an evidence-based AHRQ-endorsed care transition framework and implementation science guidelines, informing the design and implementation of our intervention [31]. This framework offers relevant guidelines for addressing challenges in discharge care transition processes and identifies contextual determinants that can inform intervention design and implementation (Figure 3). We used 5 of the 8 AHRQ-endorsed Care Transition Framework domains: intervention characteristics, organizational characteristics, characteristics and roles of clinicians, characteristics and roles of patients, and the process of implementation (Table 1).

**Table 1.** Agency for Healthcare Research and Quality–endorsed care transitions framework for the Telehealth Education: Leveraging Electronic Transitions of Care context.

Domain	Definition	Domain contextualized for TELE-TOC^a^
Intervention characteristics	Characteristics and features of the intervention that impact implementation success	TELE-TOC characteristics (TELE-TOC visit scheduling, set-up of the reminders, TELE-TOC visit content, and TELE-TOC documentation)
Organizational characteristics	Characteristics of organizations involved in the intervention, including structural characteristics, networks and communications, culture, climate, and readiness that influence implementation	UCM^b^ characteristics (insurance coverage, funding and sustainability, and ownership of TELE-TOC)
Characteristics and roles of clinicians	Attributes (eg, type of clinician, affiliations, and duties) of individuals who are engaged in the provision of care or treatment	Clinicians (medical assistant, patient service representative, pharmacist, APNs^c^, RNs^d^, etc) and their roles in TELE-TOC
Characteristics and roles of patients	Attributes (eg, type of patients) of individuals who are the recipients of care or treatment in the given intervention setting	Adult, low-acuity patients with COPD^e^ enrolled in HRRP^f^
Process of implementation	Processes including planning, engaging, and reflecting to achieve the intended use of the intervention at both individual and organizational levels	Implementation strategies for TELE-TOC

a TELE-TOC: Telehealth Education: Leveraging Electronic Transitions of Care.

b UCM: University of Chicago Medicine.

c APN: advanced practice nurse.

d RN: registered nurse.

e COPD: chronic obstructive pulmonary disease.

f HRRP: Hospital Readmissions Reduction Program.

First, we (JA and LP) followed an inductive approach consistent with our Phase 1 analysis to generate open codes about the current discharge care transition process and contextual determinants for intervention design and implementation. We then applied the constructs underlying the AHRQ-endorsed Care Transitions Framework (CTF) [32] as a structured coding template to guide deductive thematic analysis. This step helped us to align and integrate our open codes with the constructs within each of the 5 domains in the CTF as outlined in Table 1 [33]. Following this, we then formed overarching themes, or patterns across the data that related to constructs within each care transition domain, highlighting the design and implementation needs for TELE-TOC.

In Phase 3, we (JA and AM) analyzed the cognitive walkthrough transcripts using an inductive thematic analysis approach. Usability, TELE-TOC ease-of-use, and televisit effectiveness were coded and categorized into themes and subthemes (eg, barriers and facilitators). In addition, debriefing interviews were thematically analyzed to identify TELE-TOC user experiences and suggestions for improvement.

Phases 1 and 2 surveys were summarized using basic descriptive statistics (mean). Phase 3 SUS surveys had 10 questions rated from 1 to 5. In accordance with recommendations and standard practice for analysis [29,34], we subtracted 1 from each value for odd-numbered questions sharing positive statements, and we subtracted each value from 5 for even-numbered questions sharing negative statements. After summing the new values across questions, we multiplied this final value by 2.5 and then found the average score across participants.

To ensure validity and reliability, we followed best methodological practices, including member checking and team debriefing [35]. Two researchers independently coded all data, and any inconsistencies were resolved through team discussions to reach a 100% consensus.

### Ethical Considerations

This study was approved by the University of Chicago Institutional Review Board (IRB21-0947). All participants were informed of the study purpose, procedures, risks, and rights. Patients and caregivers provided written consent, and clinicians and OLs provided oral consent after reviewing the consent form with study staff. Trained personnel conducted all activities using standardized protocols, with strict confidentiality safeguards to protect participant privacy. Paper records were stored in a locked office, and electronic data were encrypted, password-protected, and housed on secure institutional servers. Hospital data were collected and stored in REDCap (Research Electronic Data Capture; Vanderbilt University), and interview data were maintained on a secure, HIPAA-compliant shared drive. Virtual sessions used the HIPAA-compliant Zoom platform with full security features enabled. Study identification numbers were used in place of personal identifiers for all analytic files. Any identifiable contact information used for scheduling or compensating participants was stored separately from study data and will be destroyed, along with audio recordings, at the conclusion of the study.

Participants in the semi-structured interviews were compensated for their time. Patients and caregivers received a US $50 gift card for each study phase they completed, and clinicians and organizational leaders received a US $15 gift card for each phase in which they participated.

## Results

### Study Overview

Forty-five participants were enrolled across Phases 1, 2, and 3 (Table 2). Findings are presented across the following themes: Phase 1: current DTOC workflow for patients with COPD enrolled in HRRP; Phases 2 and 3: considerations for TELE-TOC design and implementation.

**Table 2.** Participant numbers and characteristics by phase.

Participants	Phase 1	Phase 2	Phase 3
Clinicians	12	4	2
Pharmacist	2	2	2
Respiratory therapist	3	2	0
Advanced practice nurse	3	0	0
Nurse	1	0	0
Physician	3	0	0
Organizational leaders	5	3	0
Caregivers	2	0	0
Patients	8	6	4
Age (years), mean (SD)	63.3 (9.7)	65 (11.3)	56.3 (11.0)
Race			
Black	8	6	4
White	0	0	0
Other	0	0	0
Non-Hispanic	8	5	4
Sex			
Female	4	2	3
Male	4	4	1
Other	0	0	0

The mean (SD) patient age across phases ranged from 56.3 (11.0) to 65 (11.3) years (Table 2). Most patients were identified as Black and non-Hispanic, with no representation from White or other racial groups. Sex distribution was evenly split, with 9 female and 9 male patients overall.

### Phase 1: Current DTOC Workflow for Patients With COPD

**Figure 4.**
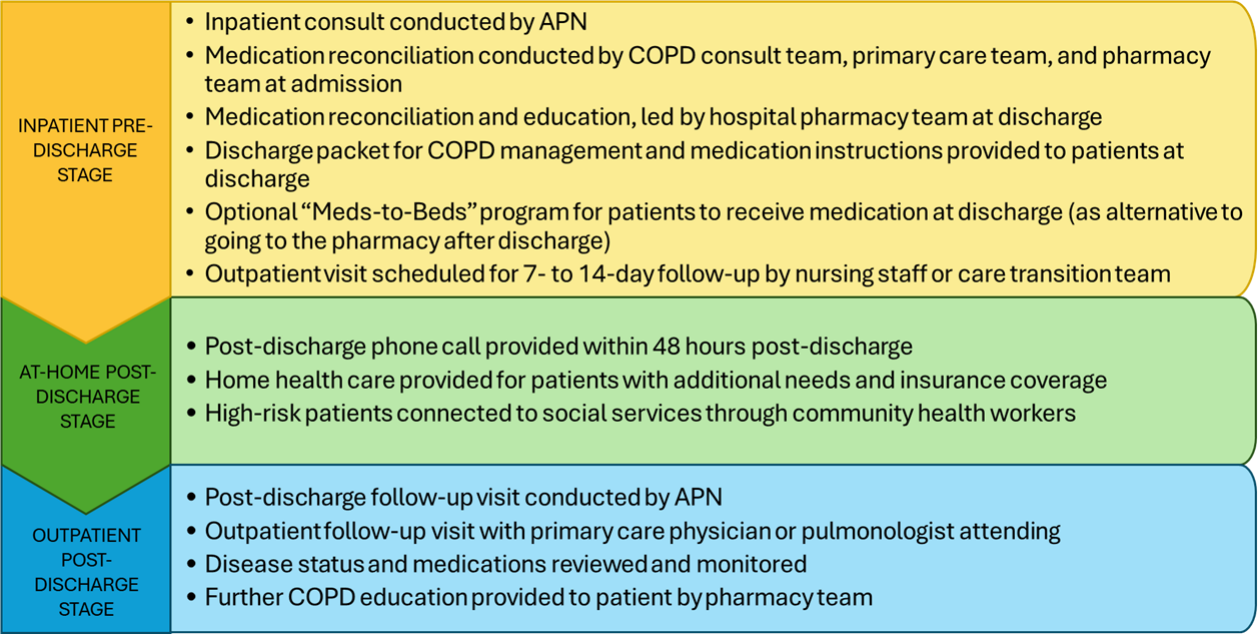
The interdependent stages of the Discharge Transitions of Care workflow. APN: advanced practice nurse; COPD: chronic obstructive pulmonary disease.

The current inpatient-outpatient discharge transition of care workflow for acute exacerbations of COPD consists of 3 interdependent stages: (1) inpatient pre-discharge (hospital admission to discharge to home), (2) at-home post-discharge (home to outpatient follow-up), and (3) outpatient visit - (Figure 4).

**Figure 5.**
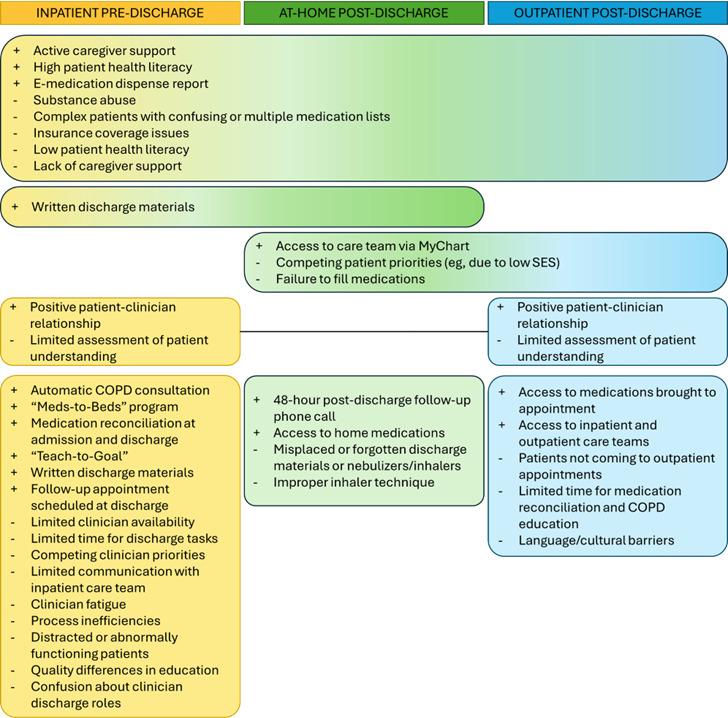
Facilitators and barriers to standard inpatient to outpatient Discharge Transitions of Care workflow. COPD: chronic obstructive pulmonary disease; SES: socioeconomic status.

All participants emphasized that each stage relied on the successful execution of activities in the preceding stage, given the interdependence in the workflow. We identified additional key facilitators and barriers to the current DTOC workflow across these stages (Table 3)—with potential impacts on safety and care continuity outcomes (Figure 5).

**Table 3.** Phase 1 topics with representative quotes. (+) Observed facilitators to current workflow; (–) observed barriers to current workflow.

Stage and topic	Quote
Inpatient pre-discharge	
(+) Medication reconciliation conducted with inpatient team	Q1. *“*[The nurses] always keep contact. Because he doesn’t have—he’s not aware of a lot of things, and we always keep everything on my phone, his charts and everything. He always tells them, ‘Ask my wife. I don’t know anything.’” [CG1^a^] Q2. “We try...talking to caregivers and involving caregivers on that discharge med reconciliation process.” [C5^b^] Q3. “I think that one of the tools that pharmacists sometimes use for medication reconciliation...It’s a button called the dispense report in Epic, which can show you what patients have picked up at local pharmacies.*”* [C11]
(+) Efficient meds-to-beds program	Q4. “Meds-to-beds was the big thing, just because pharmacies are super short right now to make sure that they got their inhalers before they are able to go home, or a bronchodilator or steroids, anything really crucial for a COPDer.” [C10]
(+) Teach-to-goal COPD^c^ education	Q5. “Our pharmacists will make sure everything’s clinically relevant...[The prescription] goes over to a technician who enters it, fills it, gets it ready. Before we deliver it to the patient, my pharmacist will do a med education over the phone with the patient. So, they call the patient, do education. Once the patient confirms they understand what they’re taking, what they’re receiving, the technician will take it and deliver it to the room. And then the patient is discharged home.” [OL3^d^]
(+) Teach-back of inhaler education	Q6. “Really, the first step is just to have them try to explain it to me...what they’re doing. And not only what they’re doing, we try to get them to verbalize why they think they’re doing it and connect those dots. So that’s the primary mechanism.” [C2]
(+) Building rapport with patients	Q7. “Part of our needs assessment when they come in on admission is asking them about their history of smoking, how many packs, and for how many years. Sometimes they won’t be as honest with you as others, that’s why you try to establish rapport with them first so they kind of open up with you a little bit better.” [C10]
(+) High patient health literacy	Q8. "What I like a lot is when...the patient’s...very well-educated, and they take care of their own health...I’ll go over the medications. They’re like, ‘Yup, I know what this is for. I take it at this time.’ And they verbally understand, and they can kind of repeat back to me, what’s going on. And they already know that their scripts are ready at home as well.” [C10]
(–) Discharge delays	Q9.“On the day that I’m supposed to be released, it was either you’re waiting for the doctors to do the final paperwork, and the nurses having to bring it together to even the pharmacy gathering up the medicines that you’ll be taking home with you to carry you until you get it going with your primary care and your pharmacist. So those were things that you just simply have to wait. And if one couldn’t do something without—the pharmacist [can’t] do their thing until the doctor does theirs. And the nurse can’t do their thing until the doctor does their thing.” [P4^e^]
(–) Insurance coverage issues	Q10. “I would say 50% of drugs that we receive require prior authorization. If it’s a prior authorization and we can’t get it approved in time or we can’t get it switched to a different therapy, we tell the patient to go home, and then we’ll work on the prior authorization.” [OL3]
(–) Absent or incomplete medication reconciliation	Q11. “The information contained (in the expense report) is limited because sometimes people will pay cash rather than use insurance or may obtain their inhalers through different sample programs or free drug programs if they haven’t been affordable to them. So, I think the incompleteness of records that people are going into those scenarios with can be a barrier and inaccuracy in existing records, and that temptation to just confirm what’s already there can be a barrier.” [C11]
(–) Low patient health literacy	Q12. “Unfortunately, the great majority of our patients don’t know their meds. And so, trying to speak to them about what is accurate and what might not be accurate on their medication list is a challenge.” [C2] Q13. “I think it really depends on the patient’s health literacy and level of understanding and...we sometimes are able to get pharmacy teaching, but we don’t always get all of those patients. So, I think they’re not always as equipped as we would like them to be [for discharge].” [C5]
(–) Poor timing of COPD education	Q14. “When a patient is getting discharged, all they think about is going home. So, we counsel them at bedside, and we give them their meds, and we teach them how to use an inhaler or whatever medication they’re receiving. But I think all they’re thinking about is going home.” [OL2]
(–) Loss of patient follow-up	Q15. "But here, once a patient is discharged, the inpatient team is 100% done with that patient. There is no follow-up.” [C2]
(–) Varied quality of education	Q16. “For patients that are started on biologic therapies that are hopefully helping them with their asthma/COPD, I think there’s sometimes confusion about whether or not they’re supposed to continue first-line therapy.” [C11]
(–) Limited knowledge of patient medications	Q17. “I think a lot of it is done by pharmacy or nursing. The physician teams are not sitting and going through medications with patients for the most part.” [OL1] Q18. “I’d say mostly just because it’s not often done by a pharmacist, and it’s done by another person who works in the institution...They don’t look to see what [the patient has] been taking, the dosage and frequencies are wrong. Routes are often wrong as well, so maybe due to lack of knowledge of some of the medications and how they’re supposed to be taken.” [C4]
(–) Time barriers and competing priorities	Q19. “The other times when med reconciliations kind of fall apart are if they get admitted in very severe illness in the MICU, they’re focused on stabilizing the patient rather than on the med reconciliation, and so sometimes...it’s not prioritized as much as it could be...” [C5]
At-home post-discharge	
(+) Active post-discharge communication	Q20. "We do have a nurse that is part of the team that does make a 48-hour phone call. And so that’s another mechanism where—if the patient actually answers the phone and is willing to engage in that conversation, that’s another opportunity for the patient to voice like, ‘Oh, I didn’t get this inhaler.’ I think she maybe reaches the patient 50 to 60 percent of the time.” [C2] Q21. “And then we also definitely found that there had to be an education component before the patient leaves the hospital, of course...I think because I think that one round of education might not be enough. And again, things might seem okay after you meet with a patient or a family, and they might feel okay about it, but then they have questions later or they forgot something.” [OL4]
(+) Use of MyChart portal to facilitate communication	Q22. “You see all them doctors I got? [sharing MyChart screen] I use [MyChart] for everything. Doctor, pharmacy, everything.” [P3]
(+) Caregiver support	Q23. “I’m thinking about one patient in particular with COPD that—and this is where the family caregivers come into play, when I need to talk to her, it’s actually through her daughter.” [C12]
(+) High patient health literacy	Q24. “...the papers given explaining about different things concerning my treatment and the issues that I have, they were pretty thorough.” [P4]
(–) Low patient health literacy	Q25. “Someone who has a low literacy, you can explain it to them, but they still wouldn’t get it.” [C8] Q26. “We do want our patients to be involved as much as they can be with their plan of care...But it is a lot for them at times...Sometimes we’ll have charts of different inhalers, because they’ll be trying to explain to us, ‘Oh, I lost this one.’” [C4]
(–) Cost-related nonadherence to COPD management	Q27. "Unfortunately, again, it’s this demographic where there is such a high amount of poor health literacy, competing priorities with patients, housing instability, food insecurity, all of those things. So taking all [medications] of this in is just not on the top of their priority list.” [C1] Q28. “I think in terms of barriers...they may have different social situations or socioeconomic factors that kind of contribute to them being able to even take care of themselves when it comes to their health.” [C5]
(–) Comorbidities impacting patient health	Q29. “Quite a few of our [patients]...with COPD are still heavy smokers, and a lot of it’s like, ‘Yeah, I still smoke,’ even though they’re on oxygen. And so that’s something else you also have to educate them on, and they’re like, ‘Well, I’ve been doing this for years. I’m okay.’” [C9]
Outpatient post-discharge	
(+) Medication review in clinic	Q30. “[In my] follow-up visit with primary care...[We go over] full medication review, how I’m feeling, [ask about] medicine I’m taking and everything.” [P7] Q31. “But sometimes people aren’t really sure which medications they are taking. I mean, the names are different, and they get changed around from time to time. And so having someone actually at home verifying, ‘Oh, these are the inhalers that they actually have at home...’” I try to have them bring in their medications.” [C3]
(+) Caregiver support	Q32. ”Also encourage them, if there is a family member or someone else that is sort of helping them with meds and kind of to keep things straight, to bring that patient—bring that person, rather, to the appointment“ [C2]
(–) Missed appointments due to travel barriers	Q33. “...it’s often not super easy for them to leave the house. A lot of them require oxygen. They’re not extremely mobile. And so actually physically getting to the pharmacy or having someone pick their medications up can sometimes be difficult.” [C4]
(–) Missed appointments due to costs	Q34. “They discuss[ed] price, like how would a medicine be obtained or any co-pays, any substitutions, or generic types. Because I was switched over to this Eliquis and more could have been explained about that.” [P4]
(–) Incomplete information/ communication with outpatient team	Q35. “Patient comes for follow-up and generally, when you’re a primary care doctor or any doctor, I would imagine, you’re sort of triaging the most important to the least important. And education and med rec tend to fall further on the bottom than direct patient care and acuity of the patient.” [OL2] Q36. “Every ambulatory clinic has a different system of how they reconcile or who reconciles or if they reconcile. So, for patients who see a lot of providers, they may or may not have a very up-to-date list.” [C2] Q37. “I think the other thing, too, is, depending on the patient’s environment...there might be a language barrier as well.” [OL3]

a CG: caregiver.

b C: clinician.

c COPD: chronic obstructive pulmonary disease.

d OL: organizational leader.

e P: patient.

### Inpatient Pre-Discharge Stage

#### Facilitators

Several facilitators enabled successful inpatient management and preparation for discharge. First, the COPD APN consultation was a mandatory step for enrolling in the COPD HRRP. In addition to clinical recommendations for the primary inpatient team, APNs provided written discharge materials to patients in the form of a COPD Action Plan. When possible, the pharmacy team provided medication reconciliation, which helped clinicians and patients to identify and address issues concerning missing or misused medications. At times, caregivers facilitated this process by playing an active role in medication reconciliation, aiding patients in recalling their home medications (Table 3, Q1 and Q2). Clinicians further noted that the Epic EHR medication dispense report aided in the medication reconciliation process (Table 3, Q3).

At discharge, eligible patients could receive additional medication reconciliation facilitated by the “meds-to-beds” program that delivered prescription medications directly to the bedside before discharge (Table 3, Q4). Finally, the pharmacy team provided inhaler instructions, using the TTG method to assess and encourage patient understanding (Table 3, Q5) [14]. Clinicians and OLs especially emphasized that using TTG at discharge significantly improved patients’ understanding of inhaler usage and follow-up care instructions (Table 3, Q6). Therefore, 67% of patients who responded in Phase 1 (4/6) felt prepared or extremely prepared to manage their COPD following inpatient discharge, with a mean (SD) survey score of 4.5 (1.03). Additionally, 83% of Phase 1 patients (5/6) agreed or strongly agreed that their inhaler education was helpful, with a mean (SD) survey score of 4.5 (0.84).

Clinicians and OLs further noted that an established positive clinician-patient relationship facilitated effective COPD education by creating rapport and improving patient comfort in speaking honestly about their needs (Table 3, Q7).

High patient health literacy also significantly aided the discharge process: both clinicians and patients felt that patients with a clearer understanding of their health were better able to retain and use COPD self-management instructions. They also felt that patients’ health literacy significantly improved medication reporting (Table 3, Q8). Finally, patients benefited from scheduling an outpatient follow-up appointment at discharge, which promoted care continuity and coordination of the next care steps with our program.

#### Barriers

Several barriers prevented a streamlined workflow and successful COPD education. Only 22% of clinicians in Phase 1 (2/9) felt satisfied with pre-discharge medication reconciliation, rating current practices a mean (SD) of 3 (0.83).

Frustrations were also reported around discharge delays due to inpatient processes that depended on multiple clinicians performing patient care tasks in a timely manner (Table 3, Q9). Another concern was inconsistent insurance coverage for “meds-to-beds.” Clinicians reported other issues with insurance, stating that time-intensive processes (eg, prior authorization and communication with pharmacies) delayed medication delivery (Table 3, Q10). While medication reconciliation was highly recommended at admission and at discharge, limited pharmacy availability during some off-hour admissions resulted in missed consultations. In cases where pharmacists were available, other complications led to a lack of clear or accurate information on patient medication, hindering medication reconciliation (eg, complex medication lists, multiple patient records, undocumented and uninsured pharmacy purchases) (Table 3, Q11). Furthermore, clinicians and OLs observed that patients with poor health literacy struggled to correctly report their home medications, often relying on caregivers to understand their medication lists and provide accurate information (Table 3, Q12). Many clinicians noted that fast-paced education at discharge coupled with poor health literacy often resulted in patients failing to retain instructions (Table 3, Q13).

A few participants noted that educating patients immediately before discharge was not the optimal time, as patients with COPD were disoriented or eager to go home, failing to engage with the discharge COPD education and medication review, and returning home confused about self-management (Table 3, Q14). Furthermore, some patients were unable to contact their inpatient team following discharge when they had questions or were unsure about self-management (Table 3, Q15). Depending on the clinician’s role (eg, respiratory therapist vs nurse vs pharmacist) and experience, the quality of patient education varied widely.

Additionally, with several inpatient team members visiting the patient and multiple lines of COPD therapy, confusion sometimes arose about instructions (Table 3, Q16). A few clinicians reported that with 6‐12 patients to manage simultaneously, challenges such as fatigue and limited understanding of roles and responsibilities in the discharge process prevented complete and successful education (Table 3, Q17 and Q18). Due to excessive clinician workloads, many stated that they frequently prioritized direct care over medication reconciliation and COPD education. As a result, only 17% of Phase 1 clinicians (2/12) felt that patients were prepared to manage their COPD following discharge (Table 3, Q19).

### At-Home Post-Discharge Stage

#### Facilitators

Despite clinician dissatisfaction, 86% of Phase 1 patients (6/7) felt that they were prepared to take their medications following discharge from the hospital, with a mean (SD) score of 4.5 (0.76). Several aspects of the DTOC workflow enabled successful COPD self-management. Patients received a follow-up 48-hour phone call from a COPD registered nurse to check in on health and address any concerns or questions (eg, obtaining medications and post-discharge visit scheduling) (Table 3, Q20). Most patients appreciated the clarification on COPD self-management that the phone call offered (Table 3, Q21). Furthermore, patients enrolled in the MyChart patient portal had easy access to communication with both inpatient and outpatient teams during transitions (Table 3, Q22). Some patients received further support from caregivers who helped with medication compliance and proper inhaler use (Table 3, Q23). Patients with higher health literacy took a proactive role in their COPD self-management and remarked that the discharge materials given to them were useful and effective (Table 3, Q24).

#### Barriers

Conversely, patients with lower health literacy or patients of lower socioeconomic status are more likely to throw their discharge instructions away or neglect to read them, resulting in improper COPD self-management. In a few cases, patients lost their nebulizers or inhalers (Table 3, Q25 and Q26). Lower socioeconomic status patients also reported competing priorities (eg, housing and food insecurity) that prevented them from focusing on COPD self-management. In addition, some could not afford the cost of their medications and therefore did not fill their prescriptions (Table 3, Q27 and Q28). Finally, clinicians reported poor patient self-management due to individual factors, including substance abuse and mental health issues (Table 3, Q29).

### Outpatient Post-Discharge Stage

#### Facilitators

At outpatient post-discharge follow-up, clinicians continued to monitor patient condition and COPD self-management. Patients were scheduled with COPD APNs for a post-discharge visit within 1‐2 weeks, where ongoing COPD care needs were evaluated and attended to; patients also received additional inhaler training from the pharmacy team. All Phase 2 patients (6/6) felt prepared or extremely prepared to manage their COPD following their outpatient follow-up, with a mean (SD) score of 4.5 (0.55). Like the inpatient setting, successful outpatient follow-ups were supported by an established relationship between the patient and the outpatient team.

Some patients were able to discuss medications more clearly by physically bringing the medications to their appointment or having their caregivers attend. This, along with access to the medication dispense report, helped clinicians to clearly understand the medications and dosages that patients were using (Table 3, Q30 and Q31).

Additional caregiver responsibilities included coordinating transportation to outpatient clinics and providing accurate descriptions of medication and inhaler use (Table 3, Q32).

#### Barriers

Clinicians reported that many patients missed their follow-up appointments for several reasons. Some could not physically travel to their outpatient follow-ups without assistance (Table 3, Q33). Furthermore, issues with insurance and medication costs continued into the outpatient stage, with patients sacrificing their follow-up appointments to pay for other necessities. Patients also reported going without medications due to expensive copays or if they were uninsured, leading to medication adherence discrepancies (Table 3, Q34).

Even though many patients spoke with their outpatient clinicians, the short appointment duration often resulted in incomplete medication reconciliation and limited assessment of patient literacy about medication use and COPD self-management. These issues, in addition to occasional cultural or language barriers, prevented clear communication and understanding between patient and clinician and sometimes resulted in misunderstandings about at-home care and medication use (Table 3, Q35-Q37).

### Phase 2: Potential Role and Impact of Televisit Intervention on DTOC Workflow

**Figure 6.**
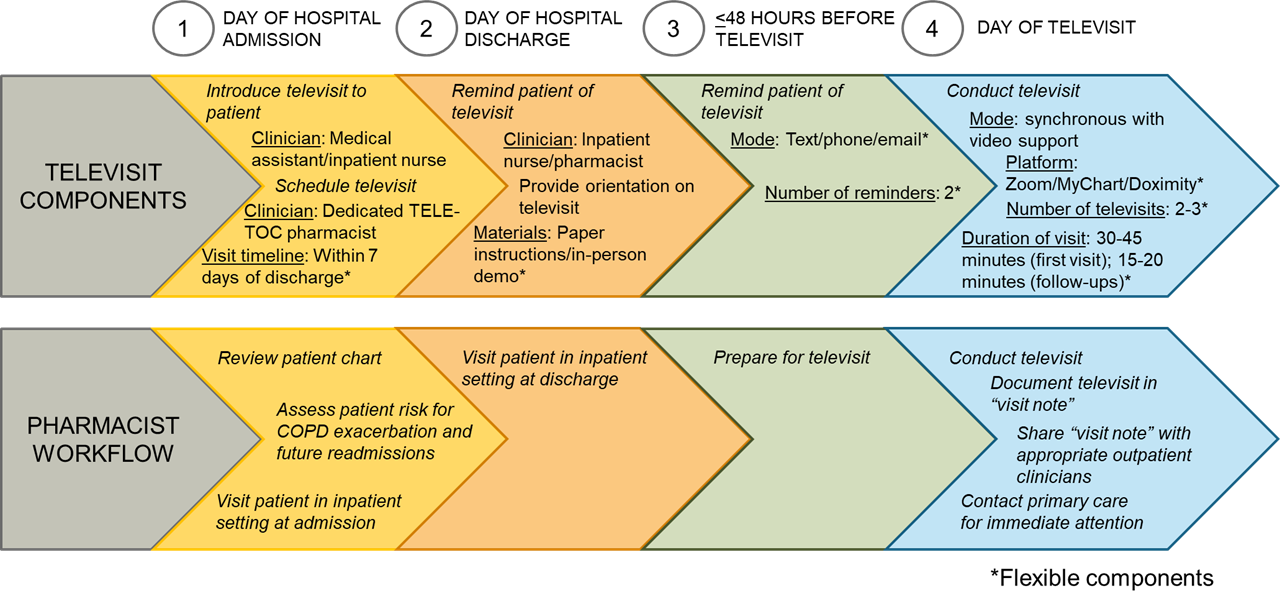
Telehealth Education: Leveraging Electronic Transitions of Care–integrated inpatient to outpatient Discharge Transitions of Care workflow. COPD: chronic obstructive pulmonary disease; TELE-TOC: Telehealth Education: Leveraging Electronic Transitions of Care.

Following our assessment of the DTOC workflow, we designed the televisit service with an initial, post-discharge, at-home televisit, followed by additional follow-up televisits before or after outpatient follow-up to maintain continuity of care. We developed a low-fidelity, televisit prototype for virtual pharmacist-led follow-up to provide inhaler training and medication reconciliation. Based on participant feedback, we categorized intervention design requirements as core (fundamental) or flexible (aspects tailored to patient preference) (Figure 6).

Participant-proposed core components included a dedicated care transition clinician role, medication reconciliation, and coordination with outpatient care teams, and COPD education to reinforce inhaler techniques and skills with teach-back. Flexible components, adapted to patient needs and preferences, would include format, structure, reminders, and platform options. Participants felt that multiple options for these components would allow for flexibility and customization based on patients’ preferences and needs. During the televisits, clinicians would use a checklist to ensure thorough inhaler education and teach-back. Additional patient COPD resources would be provided, including an inhaler use handout with visual aids and step-by-step instructions.

Most participants stated that televisits could address several barriers and augment the current DTOC workflow. They specifically mentioned potential improvements in patients’ accessibility to virtual care, which could increase outpatient follow-up adherence. Participants’ insights informed the iterative design of the televisit service, along with details on workflow and components according to patient and clinician preferences. We highlight the potential facilitators and barriers to televisit implementation (informed by the AHRQ-endorsed care transition framework) that may impact its buy-in and adoption (Table 4).

**Table 4.** Phase 2 topics with representative quotes. (+) Potential televisit facilitators; (–) potential televisit barriers.

CTF^a^ domain and topic	Quote
Intervention characteristics	
(+) Use of televisit reminders	Q1. "Maybe there’s an initial text message that you’re set for your appointment, this is your day, maybe 24 hours prior and then an hour maybe prior to their visit...Because I think that will remind a patient...like, ‘Hey, it’s time for you to get ready for your tele visit. Please sign on.’ And maybe there’s the link, and they go right to that link, and it takes them right to the visit." [OL6]^b^ Q2. “A text would be best [to remind me].” [P9]^c^
(+) Convenience of televisit	Q3. “When it be cold and they don’t have me coming out, that’s when I do it on the phone with the doctors...Yeah. And raining. I ain’t going out in the rain.” [P11] Q4. “We’ve actually seen an increase of [patients] attending visit(s) when it was telehealth because they didn’t have to worry about getting a ride or commuting here.” [OL3]
(+) Flexible televisit timing and frequency	Q5. “[Wait to schedule the visit for] at least about a week...And that way, they...try to get familiarized with their house again because...coming home from the hospital is an experience.” [P14] Q6. “I think most of the time, patients are still overwhelmed when leaving the hospital...I think the sooner, the better to ensure that they start off on the right foot as far as utilizing their medications appropriately to prevent readmission.” [C16]^d^ Q7. “I think two to three visits would be reasonable...” [C15]
(+) Convenient documentation and education via televisit	Q8. “One of the cool things about telehealth is you can actually have someone show you. So as long as they were doing it through a cell phone or a laptop or was mobile, I would say, ‘Okay. Show me where you’re storing your medications.’ But through having patients show me their meds, I had a checklist of...is it being stored at the correct temperature, right? Is it in their fridge or not? Is it being stored mindfully with regard to if it’s something that shouldn’t be exposed to light?” [C11] Q9. “I think the first visit is going to be primarily teaching, me showing them, ‘This is how it’s done, essentially.’ And then second and third visit would be more assessing, ‘Now that you’ve learned how to do this properly, how are you actually doing it?’ And then additionally, outside of even inhaler technique, "Are you remembering to take it every day? How can we do those adherence sort of things, like setting reminders on your phones, engaging family members to help you, things like that?” [C13] Q10. “In the first half, making sure there’s no contraindications or medication, like disease and drug interactions. I always document that it’s a virtual visit. And I also document if an interpreter or translator is needed. In the second half, I talk about medications: ‘Are you still taking amlodipine? What are you taking for blood pressure...What medications are you taking?’” [C14]
(+) Dedicated role to support patients post-discharge	Q11. “Just need the support (for a patient), either over the phone or in person to help navigate.” [P9]
(–) Patient lack of access to technology	Q12. “You want to make sure people have a camera, obviously, to use the video visits...So, getting a webcam. Making sure you either have headphones or a speaker or some way that you’re going to physically communicate because a lot of the webcams may have speakers but may not necessarily have a microphone to pick up sound.” [C15] Q13. “I mean, there’s plenty of barriers. Do they have phone service? Do they have cell service? Do they have internet access? Are they able to use Zoom or other video-conferencing software or MyChart software to access the visit?” [C5]
Characteristics of clinicians	
(+) Clear roles and responsibilities for clinicians	Q14. “I think you also need a PSR to schedule them...It’s a big burden to worry about constantly scheduling the patients. What we do is we provide the schedulers with a template of the times where we are available, and then the schedulers place patients on that schedule.*”* [C14] Q15. “I think the person that probably has the least time (to conduct a tele-visit) is probably the MD...It’s hard to say. My mind drifts towards RN maybe having more time.” [C13]
(–) Lack of clinician bandwidth	Q16. “One big barrier for myself has kind of been a theme all along...doing the notes was a barrier for me. These notes...were relatively in-depth in that they were kind of like progress notes...But I think that was a major barrier for me in that if the average visit was 45 minutes and I had 60 minutes allotted to it, that meant I had to write my note in 15 minutes, which wasn’t always possible. And then sometimes staying late taking work home.*”* [C13]
(–) Inability to conduct in-person physical assessments	Q17. “Some patients feel like it’s not a good visit, like it’s not a real visit because we didn’t touch them or really get to see them or do full vitals.*”* [C1]
Characteristics of patients	
(+) High patient health literacy	Q18. “[I want to learn about] my health, medications, the do’s and the don’ts.” [P9] Q19. “I do think you’re right. I think that there are some patients who you could probably do this in 10, 15 minutes, and it just goes by really quickly, and other patients that are going to take 45.*” *[C3]
(–) Low patient health literacy	Q20. “Oftentimes, our patients have a gap in health literacy which impedes some things, which this would be a nice bridge to. But there would be also the concern that their technical literacy might also prevent them from doing a visit*...”* [OL4]
(–) Low patient technology literacy	Q21. “What is their, I guess you can say, digital literacy? Do they know how to use it? Do they know how to get on to that?...When I think about the population that are most likely COPD, it’s usually an older population. I don’t know if they understand how to use that technology.*”* [OL3]
Process of implementation	
(+) Provision of training strategies for patients	Q22. “So, I mean, if we’re doing these visits through like a telehealth type visit...maybe it’s techniques of how to be able to engage with a patient through a Zoom type of link and different techniques to make sure that you can assess the patient is able to do the proper techniques if they’re using an inhaler or something.*”* [OL6]
(+) Back-up strategies for implementation	Q23. “Usually, we’ll just ask the patient if we can call them on their cell phone. So that way, at least if there’s anything major that we can still conduct most of the visit and still address anything that’s immediate.*”* [C16]
(+) Use of hands-on teaching strategies	Q24. “I ask them to show me basically how do you do it, without actually doing it. And if they don’t, then what I usually try and do is, if there’s a video, I’ll show them that, because I think that’s really nice and typically, they can explain it better than I can. And then I’ll supplement that with, “Okay. What questions do you have? And kind of show me what they showed you,” and kind of do hands-on teaching from there.*”* [C13]
(+) Incorporation of buy-in strategies	Q25. “Yeah. And then you make sure that their inner (support) circle...knows [about COPD management].*”* [P14]
(–) Health care hierarchy clashes	Q26. “I think doing the telemedicine at the skilled nursing facility is...really difficult. And the reason...is because they basically have somebody there giving them the medication...And theoretically, it’s a trained professional who should be watching them do it. So I think that’s difficult. Are you kind of stepping on someone’s toes by doing it there? I don’t know.” [C13]
Organizational characteristics	
(–) Insufficient funding	Q27. “I think that you’re going to want to think about how...if the program is successful, how are you going to sustain it? A lot of these things that—it’s great. They get grant funded. They get developed. And then once the grant funding runs out, then sustainability becomes an issue.” [OL1]
(–) Lack of TELE-TOC^e^ ownership	Q28. “One of the things we had to figure out was who’s going to own this process? Was it going to be nursing? Was it going to be my team? Was it going to be ambulatory? And so, coming up with that solution, I think, is one of the initial steps.*”* [OL5]

a CTF: Care Transitions Framework.

b OL: organizational leader.

c P: patient.

d C: clinician.

e TELE-TOC: Telehealth Education: Leveraging Electronic Transitions of Care.

### Intervention Characteristics

#### Facilitators

All participants felt that televisits could be supported with 2‐3 text or phone call reminders to increase attendance and effective usage. While most were flexible with reminder timing, some specifically requested a reminder the day before or on the day of the televisit. Patients who preferred text reminders also felt that helpful messages should include tele-visit details (eg, clinician information, timing, and connection instructions) (Table 4, Q1 and Q2). Several patients believed that clarification for questions and concerns was more easily addressed via televisit between discharge and outpatient follow-up, allowing easy access to a pharmacist in the comfort of their homes (Table 4, Q3 and Q4). All patients and most clinicians and OLs believed that 2‐3 televisits would be optimal (Table 4, Q7). They also felt that patients needed to settle in at home, observe treatment effectiveness, and gather any questions or concerns they have regarding medication use or discharge instructions, so televisits should be scheduled at least a week past discharge (Table 4, Q5 and Q6).

A few clinicians highlighted that using video televisits allowed patients to more easily show pharmacists their medications and inhaler routines (Table 4, Q8 and Q9). Most clinicians and several OLs also felt that the televisit service could be facilitated with the Epic EHR visit note template to document patient information, medication regimen, adverse events, quality of life, and patient counseling. In the event of an emergency, this template could be forwarded to other clinicians. Clinicians stated that documentation could reduce information omission and maintain consistency (Table 4, Q10). Additionally, clinicians suggested that visits should be led by a nurse or pharmacist, as they were knowledgeable about their patients and often had more availability. Some patients also believed that televisits could be more effective with technical support (Table 4, Q11).

#### Barriers

A few clinicians and OLs highlighted that a fraction of patients might not have internet access, limiting their ability to connect to the televisit. Furthermore, issues with internet connectivity or video quality could prevent clinicians from seeing patients and medications clearly (Table 4, Q12 and Q13).

### Clinician Characteristics

#### Facilitators

To avoid confusion about responsibilities and promote successful televisit implementation, most clinicians felt that a medical assistant, patient service representative, or nurse should schedule televisits. Many also recommended that clear roles and responsibilities be outlined to ensure better coordination and improvement of patient outcomes (eg, medical assistant or patient service representative for scheduling, nurse or pharmacist for televisit) (Table 4, Q14 and Q15).

#### Barriers

Several clinicians mentioned that time and workload could significantly limit their ability to conduct and document televisits. They worried that extra time after the visit would be required to accurately document the session, increasing the workload and potentially affecting documentation quality (Table 4, Q16). Others also stated that they might struggle with assessing physical symptoms through virtual televisits, impacting patient satisfaction (Table 4, Q17).

### Patient Characteristics

#### Facilitators

Several clinicians felt that patients with high health literacy would be the most proficient users of the televisit service, as they would be more likely to engage during COPD education (Table 4, Q18 and Q19).

#### Barriers

Some clinicians and OLs worried that poor patient health literacy or technology literacy could negatively impact patient engagement during the visit, limiting the effectiveness of televisit outcomes (Table 4, Q20 and Q21). A total of 50% of Phase 2 patients (3/6) stated they had never used video technology platforms or conducted televisits, and several were not comfortable with FaceTime (Apple, Inc), WhatsApp (WhatsApp LLC), or other video chat technologies, scoring their comfort a mean (SD) score of 2 (2.00). Furthermore, some clinicians predicted that patients might have difficulty using apps such as Zoom videoconferencing software or the MyChart patient portal. Only 1 patient respondent was 100% comfortable using the internet, and many stated that they would need help, with a mean (SD) score of 2.3 (1.63).

### Process of Implementation

#### Facilitators

To successfully implement the televisit, participants all suggested training and education for both clinicians and patients on the visit procedure (Table 4, Q22). A few clinicians and OLs also suggested conducting televisits via phone call if technical issues occurred (Table 4, Q23). Several clinicians believed that using demonstrational videos or other visuals would help to engage patients during televisits, increasing their understanding of COPD management and improving patient outcomes (Table 4, Q24). To encourage buy-in, a few clinicians and patients suggested involving caregivers in the televisit. As caregivers played an active role in patients’ lives, participants speculated that they could encourage treatment adherence and aid the medication reconciliation process (Table 4, Q25).

#### Barriers

Several clinicians mentioned that some patients might receive care at skilled nursing facilities, which could introduce patient management conflict between tele-visit clinicians and in-facility clinicians (Table 4, Q26).

### Organizational Characteristics

#### Facilitators

Our participants did not report any organizational facilitators within our data.

### Barriers

All clinicians and some OLs strongly felt that secured funding for and clear, defined ownership of the televisit service were essential for long-term sustainability and consistent clinician accountability (Table 4, Q27). A small number of clinicians also highlighted their lack of dedicated space to conduct televisits, potentially making both the clinician and patient uncomfortable and affecting the quality of communication (Table 4, Q28).

### Phase 3: Participant Perceptions of TELE-TOC Prototype

Following 5 walkthroughs with our high-fidelity TELE-TOC prototype, patients and pharmacists discussed their perceptions of TELE-TOC and identified facilitators and barriers to TELE-TOC overall, medication reconciliation, and inhaler education. Furthermore, suggestions for improvement were made (Table 5).

**Table 5.** Phase 3 topics with representative quotes. (+) Observed facilitators to Telehealth Education: Leveraging Electronic Transitions of Care; (–) observed barriers to Telehealth Education: Leveraging Electronic Transitions of Care.

Theme and topic	Quote
Participant perceptions of TELE-TOC^a^	
Appropriate content and duration of TELE-TOC sessions	Q1. “I still have some questions too. And with the pharmacists too, we could sit down and we could talk a little bit more. I figured we didn’t talk long enough.” [P16]^b^ Q2. “[The tele-visit] gave you all the—it gave me all the information I needed to help me, like I said, because I have to help me, not just let somebody else help. So that’s what I like about this.” [P17]
Preference for hands-on engagement	Q3. “Just it was helpful to have someone actually really explain it rather than just hand you a piece of paper and your meds and say, ‘Here you go.’” [P16] Q4. “[The inhaler instruction] was easy because I can actually see the way he was showing me how to do it because I’m a visualist. I need to be able to see you do it so I know, ‘Okay. This is how I need to do it.‘ And that helped me.” [P17]
Improved confidence in COPD self-management	Q5. [Upon being asked by the clinician whether the tele-visit made the patient more confident in COPD self-management] “Yes, because I know as long as I do what I’m supposed to do, my chances of going to the emergency room are decreased. I prefer to be able to—like you said, I’m in the comfort of my own home...I just want to have everything that I need right here with me and it’ll be accessible to me so I can take care of me better.” [P17] Q6. “For me, it’s just like a sparkle that somebody even cared enough to talk later...You came in there—you came in there and talked to me, and I feel like we’ve been knowing each other for—do you understand what I’m saying?” [P18]
Experiences with medication reconciliation component of TELE-TOC	
(+) Straightforward medication reconciliation with use of video technology	Q7. [Upon patient confusion about two medications with the same cover but different names] “No, no, no, no, no. We got the Symbicort. I told you I had two of them. It was only one...Can you see that?” [P16]
(+) Easy access to patient health information via MyChart	Q8. [Upon clinician asking if the patient was taking Lasix] ”I was taking some Lasix. And I was just told to leave that alone too. So I’m not taking Lasix anymore...They cut that out.” [P16] Q9. [Upon clinician asking if the patient received azithromycin recently] “No, I don’t even know what that is.” [P16]
(+) Improved understanding of medication through in-depth patient education	Q10. “When you give somebody or tell somebody to take stuff, tell them what it’s for and make sure they understand what it’s for. Just don’t [say] like, ‘Here, take this here three times a day and call me next week and let me know how you feel.‘” [P16] Q11. [Upon patient asking about what a spacer does in relationship to inhalers] “Oh, that’s not for your inhalers. That helps strengthen your lungs. So that helps kind of control—so when you push this button, the medication comes out really quickly. This allows it to just kind of—allows us to take a much easier and deep breath with the medicine, and it allows it to get fully into our lungs.” [C17]^d^
(–) Lack of patient preparation for medication reconciliation	Q12. “It would have been better if I had a copy of that [medication] list to go over it with you. [When asked if the patient could find their medications to show the clinician] I can’t find them right now. That’s what I was looking around for, but I was getting short of breath just looking for them.” [P16]
Experiences with inhaler education component of TELE-TOC	
(+) Enhanced inhaler education through visual cues from video technology	Q13. “So what I’m going to have you do is you’re just going to act. As we go through this, you’re just going to act like you’re taking it. I’m not asking you to take an extra dose. But as we go through it, I want you to show me what you would normally do. So from the beginning, and don’t open the lid all the way, that’ll waste a dose, but we’ll act like the lid is open.” [C18]
(+) Improved understanding of inhaler education through teach-back method	Q14. “I’m going to go ahead and show you really quickly. I’m just going to show you the steps and how I would use the inhaler and then have you show me one more time on how you go ahead and use it, okay?...[Upon explaining the steps and demonstrating inhaler use] That’s all I would do...Can you just go ahead and show me one more time how you would do it?” [C17]
(–) Lack of patient understanding despite education	Q15. “[My confidence in my inhaler use is] still up in the air because I don’t know what triggers it. I don’t have a full understanding of my concentrators, of how they’re working and what to look for or when do I need to change the hoses on that and stuff. So there is still a lot more to be done with this topic.” [P16]
TELE-TOC use and implementation	
(+) Smooth TELE-TOC process	Q16. “Yeah. It was not something that I had to think hard about. It was easy for me to just go with the flow. I didn’t have to struggle trying to understand, “Okay. What are they going to ask?” I just went with it, and everything flowed right through naturally for me.” [P17] Q17. “In terms of the last one, I think, honestly, overall, the flow was a little bit better. Obviously, this patient didn’t have their medications, but that is real world. But I think overall, say we were able to do the full visit top to bottom...” [C17]
(+) Convenient use of video technology	Q18. [Interviewer asks whether the order of the tele-visit was easy to follow] “Yeah. Because I [inaudible] I see it.” [P15] Q19. “[The tele-visit was] Great. I don’t have to come out. [laughter] I kind of struggle with [leaving the house]. And then I got anxiety. So, the fact is, when I can’t breathe, I panic, and it’s bad.” [P15] Q20. “I felt good because I’m comfortable here. I didn’t have to come out into the cold or sitting in an office where I’d be uncomfortable. I’m comfortable sitting here. I’ve got my oxygen on too, so. But it felt good to be able to be at home and do something like this.” [P17]
(+) Helpful reminders for sessions	Q21. “I appreciated [the reminder]. Yeah, it was very helpful because sometime I will forget some stuff. Then this morning they sent me one at 7:47 to say, ‘It’s time to log in.’ So I was able to log on in.” [P17]
(–) Patient desire for more in-depth education	Q22. “I’m concerned about it all. And I need someone to be there to go over all of it. What should I do? If there’s something should happen, what should I do? I don’t want to be—I don’t want to be the one doctoring on myself.” [P16]
(–) Technical issues with Zoom	Q23. “I can’t hear them. Can’t hear anybody.” [P16] Q24. “My phone is not muted. My phone is not—hold on. Let me see. Microphone, iPhone microphone. We have your audio. I don’t know why you can’t hear me.” [C18]
(–) Low patient technology literacy	Q25. [When asked about difficulty of logging into MyChart for tele-visit] “Usually, it’s really hard for me to get in, and I just give up. But it really wasn’t that bad. I guess I needed the hands-on to show me how to do it.” [P15]
(–) Confusing “visit note” for pharmacists	Q26. “[It was confusing inputting information into the “visit note”]. Specifically under the section, quote, ‘script for pharmacist.‘ It just doesn’t feel like this necessarily belongs in a ‘visit note.‘ I’ve never seen an outpatient note say, ’This is your script that we want the doctor to say or the pharmacist to say, and then these are the responses.‘ So I just find it difficult to know what’s supposed to be included there. I’ll include his answers, but his answers are also included two or three other times in the note. For example, the question, ‘What medication do you take at home?‘ And then further down, it has a list of his medications. So I don’t really know. Do I type every medication there? It doesn’t make sense.” [C18]
Suggestions for improving content and implementation	
Ask patients to prepare medications prior to sessions	Q27. “Right. And then I would like to have— like to know to have all my medicines with me that I’m taking so that we can go over them. Don’t wait till I get on the meeting and say, ‘Dude, where’s your medicine? You got this. You got that.‘ You have all your medicines with you before we start this meeting. And that way, you got my undivided attention.” [P16]
Provide detailed education on COPD	Q28. “[I’d like to know more about...] Maybe the causes of COPD. And how it could be treated. [Clinicians] usually just give you a paper and tell you to read aftercare, but I think if you talk about it more, people will understand it more.” [P15]
Provide clearer inhaler instructions at discharge	Q29. “The [first day] I made it to a visit anywhere and somebody gave me an inhaler, we should have went over what we went over today. That should have been from day one...Anything that’s being used to help you breathe, it should be an in-depth— get a video of something that you should watch. All right? I mean, okay, a video is something. It’s a start.” [P16]
Provide a tutorial for TELE-TOC video chat	Q30. “It probably would take maybe some training for them, or I would say if they were going to participate, don’t you know how you came in the room with me before we—you probably want to just ask them if you can see their phone and make sure the Zoom is set up on there.” [P18]
Amend “visit note” for streamlined sessions	Q31. “Probably, just the template of the note I would reorganize a little bit because it kind of goes from med rec to COPD med rec and then—or COPD meds, and then it goes back to med rec again, which definitely threw me off. And there’s a lot of noise in the note, so it’s kind of hard to—while you’re trying to look at a patient, look at a note, but then also conduct something, it’s a lot to go back and forth. So I think probably rearranging the note a little more will be pretty beneficial by kind of just grouping med rec and then all the COPD stuff itself.” [C17]
Conduct TELE-TOC in a group setting	Q32. “I would like to do it as a group. I’d like to have more than one, besides me, I want to have another patient there with me also. So we can compare our notes together. Me and that patient can talk. And that way, you’ll get a better understanding [of] how we feel...” [P16]
Target patients with medication needs	Q33. “I think we were able to still go through the entire visit, identify some things that the patient can do better from their inhaler standpoint and then also how we can go ahead and assist the patient in terms of their medicines, not necessarily with adherence because we can’t dive too much into that, but in terms of medicines they need. Medicines that they’re out of and they need refills and stuff like that and also duplicate therapies.” [C17]

a TELE-TOC: Telehealth Education: Leveraging Electronic Transitions of Care.

b P: patient.

c COPD: chronic obstructive pulmonary disease.

d C: clinician.

### Participant Perceptions of TELE-TOC

Patients and pharmacists described an overall positive experience with TELE-TOC. Patient SUS scores were reported as an average of 97.5/100, indicating that patients found TELE-TOC to be user-friendly and easy to use. Furthermore, when asked to rate the overall quality of TELE-TOC on a Likert scale from 1 (very poor) to 5 (very good), on average, patients rated TELE-TOC 5/5, while pharmacists rated TELE-TOC 3.8/5.

Most participants felt that TELE-TOC was not difficult to engage with and even suggested lengthening the sessions to create more time for questions and patient-specific concerns (Table 5, Q1 and Q2). When asked about TELE-TOC content, patients responded that they did not feel that any content needed to be removed; on the contrary, they requested the addition of more in-depth COPD information. Pharmacists and patients agreed that TELE-TOC was engaging and helpful, especially the inhaler education, which patients described as more hands-on than their discharge education and in-person follow-up visits (Table 5, Q3 and Q4). Patients stressed that TELE-TOC’s follow-up helped them to feel cared for and improved their confidence in medication management and inhaler use (Table 5, Q5 and Q6). They also felt that TELE-TOC motivated them to learn more and improve their COPD management.

### Experiences With Medication Reconciliation Component of TELE-TOC

#### Facilitators

Regarding medication reconciliation, a pharmacist noted that it was easier to confirm which medications each patient was taking based on what they presented on video (Table 5, Q7). Furthermore, use of MyChart allowed pharmacists to easily access medication lists to double-check medication names, dosages, and frequency of use (Table 5, Q8 and Q9). Through TELE-TOC, pharmacists could identify prescription discrepancies while helping patients to better understand the purpose of each medication (Table 5, Q10 and Q11).

#### Barriers

Challenges with medication reconciliation included lack of patient preparation and low patient health literacy. Pharmacists noted that patients were not always adequately prepared to review their medication lists and may need reminders to have their medications ready for review in future sessions (Table 5, Q12). Some patients also simply read their medication labels but were unable to fully understand their medication management, even with help from the pharmacists.

### Experiences With Inhaler Education Component of TELE-TOC

#### Facilitators

During inhaler education, video technology enabled pharmacists to see how patients used their inhalers and instruct them accordingly (Table 5, Q13). Most patients agreed that video technology made instructions easier to follow through visual feedback. They also enjoyed the teach-back method used by pharmacists, noting that it felt more engaging and helped them process and understand clinician instructions (Table 5, Q14).

#### Barriers

While most patients felt satisfied with their inhaler education from the TELE-TOC pharmacist, others reported that the lack of further in-depth education left them with an incomplete understanding of how to use an inhaler (Table 5, Q15). They felt that even with the education provided during the session, they were still not fully confident in their inhaler use.

### TELE-TOC Use and Implementation

#### Facilitators

Overall, patients noted that TELE-TOC’s flow was easy to follow and led to a more streamlined process of medication reconciliation and inhaler education (Table 5, Q16). Both pharmacist interventionists agreed that both components of TELE-TOC were easy to complete smoothly, and TELE-TOC did not require too much extra work to conduct (Table 5, Q17). Using video technology also allowed patients to speak to their pharmacists from the comfort of their home. Unanimously, patients agreed that the telemedicine nature of the intervention allowed for improved access to their clinicians and increased the likelihood of attending the virtual session, in comparison to a similar in-person follow-up (Table 5, Q18-Q20). Additional facilitators to TELE-TOC implementation included patient reminders via MyChart to ensure patient attendance (Table 5, Q21), as well as technology literacy among pharmacists to correctly set up visits. Both pharmacists noted that they did not have issues with navigating TELE-TOC or MyChart.

#### Barriers

Patients and pharmacists listed different barriers to TELE-TOC implementation. Several patients believed that TELE-TOC was helpful for those who were unfamiliar with their medications or inhalers, but others voiced the desire to learn more about their COPD and management beyond the general information provided alongside medication and inhaler education. For example, some patients wanted to better understand COPD epidemiology, along with signs and symptoms to watch out for at home; lack of further in-depth discussion about their condition and experiences left them feeling mildly dissatisfied (Table 5, Q22).

Technology literacy and technical issues were observed as impediments to conducting TELE-TOC. Over multiple sessions, technical issues within Epic EHR and over Zoom made TELE-TOC difficult to conduct (Table 5, Q23 and Q24). Further, while patients stated that they did not have too much difficulty or concern with navigating the technology required to conduct TELE-TOC, issues such as forgotten passwords, confusion about MyChart login information, and misunderstandings about Zoom settings were observed during walkthroughs. One patient even noted that without technology assistance from the clinician or a caregiver, they would likely have given up on TELE-TOC (Table 5, Q25). These issues were quickly resolved with the help of TELE-TOC pharmacists, who were trained to provide several solutions to technical barriers (eg, switching to telephone calls, moving from MyChart to Doximity, and providing clear instructions in the event of patient problems with log-in or calling).

Both pharmacists also provided feedback on the TELE-TOC workflow and tools, primarily voicing initial dissatisfaction with the “visit note.” They initially felt that the “visit note” was not structured in a way that allowed for streamlined conversation and were unsure of the needed depth for the notes (Table 5, Q26). As both pharmacists conducted more walkthroughs, they verbalized that the structure and clarity of the “visit note” greatly improved over time.

### Suggestions for Improving TELE-TOC Content, Function, and Implementation

Following the discussion of the barriers to their TELE-TOC experiences, participants provided a few suggestions to mitigate these challenges. To combat a lack of patient preparation for and to streamline medication reconciliation, it was suggested that pharmacists ask patients to preemptively prepare their medications prior to TELE-TOC (Table 5, Q27). Furthermore, more detailed education and anticipatory instructions on inhaler use and general COPD management were suggested as a method to support patients who still felt unsure in how to care for their conditions (Table 5, Q28 and Q29).

Participants also felt that providing clear instructions and a tutorial on how to use Zoom and MyChart technology would be helpful in assisting patients with lower technology literacy (Table 5, Q30). Furthermore, amendments to the “visit note” were suggested: sequence the note in alignment with the structure of each session and provide clearer instructions for pharmacists to complete the document (Table 5, Q31). Other suggestions included establishing an additional group format of TELE-TOC to provide inhaler and COPD education for multiple patients at once (Table 5, Q28), as well as using TELE-TOC as an additional source of support for patients with medication needs, such as checking on refills or checking for duplicate therapies (Table 5, Q32).

## Discussion

### Principal Results

Our 3-phase, UCD approach tailored and optimized an evidence-based televisit medication management and education intervention for patients with COPD transitioning from hospital to home. We identified facilitators, barriers, and interdependencies across the current DTOC workflow for patients with COPD, seeking to amend the workflow and improve patient outcomes. Key facilitators across settings included the “meds-to-beds” program and caregiver support, while challenges to effective care included inopportune education timing, inaccurate medication records, and limited outpatient follow-ups. These findings suggested the need for more comprehensive post-discharge patient support, which indicates the utility of the TELE-TOC intervention.

While the study was open to patients of all races and ethnicities, all enrolled patients identified as Black and non-Hispanic. This aligns with trends observed in UCM’s COPD HRRP, in which a large proportion of eligible patients are Black. As a result, our study sample reflects the demographic makeup of UCM’s patient population. Several factors, including lower utility of health care services, health insurance coverage, and behavioral differences (eg, smoking habits), have increased COPD’s impact on Black patients [36,37]. Hence, Black patients hospitalized with COPD exhibit greater symptom severity and have significantly higher odds of 30-day readmission in comparison to non-Hispanic White patients [38-40]. Despite this disproportionate effect, very few studies examining the implementation and effectiveness of COPD DTOC interventions have explicitly included Black populations [41].

### Comparisons With Prior Work

Our study corroborates existing literature, which emphasizes clinician COPD training and positive patient-clinician relationships as key facilitators to successful post-discharge care, alongside key barriers such as resource constraints, limited patient understanding of the education provided, and inappropriate allocation of tasks among clinical staff [17,42,43]. However, we identified additional barriers not widely discussed in prior research, such as patients using cash online pharmacies, resulting in undocumented medication use. This underscores the importance of accurate medication reconciliation, which is often limited in hospital settings.

We then explored how to address these barriers using pharmacy-led, virtual, in-home televisits for medication reconciliation and inhaler education. Our data suggested that televisits would be well-received and engaging due to their convenience and the addition of a dedicated pharmacist during the critical care transition period. Prior literature also highlighted the significant advantages of telehealth in medication management and virtual health education. For example, a previous study found that patients benefited more from telehealth (vs usual care) medication reconciliation, showed higher engagement and buy-in, and maintained better adherence to prescribed medications [44]. Further, multiple reviews [24,45,46] noted that telehealth-based interventions have improved patients’ post-discharge healthcare outcomes and quality of life.

Based on these insights, we refined the TELE-TOC components and workflow. Notably, TELE-TOC offers a customizable telehealth experience that tailors televisit format (eg, visit mode, dose, frequency, and structure) to meet patient preferences and clinical needs, enhancing engagement and ensuring more personalized care while retaining core patient discharge components. Both patients and clinicians found TELE-TOC effective and efficient for medication reconciliation and comprehensive inhaler education. Despite some patients suspecting they would struggle to log in and navigate to TELE-TOC on their own, SUS scores remained high due to support and clear instructions from clinicians. Furthermore, due to a 766% increase in telemedicine encounters following the COVID-19 pandemic [47,48], many patients and providers report improvements in their televisit competency, which could explain the high success rate of TELE-TOC among our participants. Apart from a few technical difficulties, both patients and providers noted positive experiences with TELE-TOC usability and effectiveness [49]; other studies have reported similar feasibility and usability of eHealth interventions for patients with chronic conditions [50]. Other key success factors included remote monitoring and feedback from clinicians, visual aids via videoconferencing tools, and access to medication lists at home. Use of previously successful implementation strategies, such as patient-personalized televisit format, inhaler education teach-back, and TTG methods, was crucial to the success of our TELE-TOC prototype [13,16,51]. Several implementation barriers were addressed, including clinician availability for COPD education and patient forgetfulness about medications [52-58]. However, structural barriers, such as patient financial burden related to payment structures, remain unaddressed [52,59-61].

To evaluate the clinical impact of TELE-TOC, we are currently conducting a randomized controlled trial evaluating the effectiveness and implementation potential of TELE-TOC compared to usual care at UCM (NCT06461403), which is expected to be completed in 2026 [62]. Our primary effectiveness outcome assesses inhaler technique at 30 days after discharge, based on a validated 12-step checklist. Secondary outcomes include acute care revisits, rehospitalizations, and mortality rates at 30, 90, and 180 days post-discharge, in addition to medication errors, changes in COPD symptoms based on the COPD Assessment Test and the Modified Medical Research Council dyspnea scale, and inhaler technique. Our primary implementation outcome is intervention reach. If successful, TELE-TOC could serve as a scalable model for improving COPD discharge care transitions and reducing avoidable acute exacerbations of COPD readmissions.

### Limitations

We acknowledge our study limitations. First, this is a single-site study at an urban academic medical center, which may limit generalizability. In particular, the racial homogeneity of our study sample may limit the generalizability of our findings to the broader US population. Nevertheless, this study attempts to address the research gap in developing COPD discharge care transition interventions to meet the needs of Black patients. Second*,* we acknowledge that within our small sample for Phase 3, some patients mentioned familiarity and experience with using phones and computers to perform tasks, including video calls. This experience with technology may strongly contribute to their usability ratings, and we acknowledge that our SUS findings may reflect limited sample diversity. However, as previously mentioned, televisit proficiency increased significantly during the COVID-19 pandemic, and we anticipate that within our current randomized controlled trial, with a larger, more diverse sample, we may see similar TELE-TOC proficiency among our target population. Third, the feasibility of televisit interventions may not be applicable across health care organizations, depending on institutional resources. To best address this, we will develop an evidence-based roadmap for pragmatic implementation and dissemination of TELE-TOC across diverse rural, urban, and suburban US hospitals.

### Conclusions

We adopted a UCD approach to codevelop and optimize a pragmatic and patient-centered COPD televisit intervention to support hospital-to-home care transitions. The goals of the proposed TELE-TOC intervention were to reduce unnecessary readmissions and improve continuity of care, provide impactful medication management and education to improve outcomes such as COPD symptom control, and ascertain implementation outcomes, including reach, sustainability, and scalability of TELE-TOC.

## Supplementary material

10.2196/77953 Multimedia Appendix 1 Telehealth Education: Leveraging Electronic Transitions of Care interview guides.

## Abbreviations

AHRQ: Agency for Healthcare Research and Quality
APN: advanced practice nurse
COPD: chronic obstructive pulmonary disease
CTF: Care Transitions Framework
DTOC: Discharge Transition of Care
EHR: electronic health record
HIPAA: Health Insurance Portability and Accountability Act
HRRP: Hospital Readmissions Reduction Program
OL: organizational leader
SUS: system usability scale
TELE-TOC: telehealth education: leveraging electronic transitions of care
TTG: Teach-to-Goal
UCD: user-centered design
UCM: University of Chicago Medicine

## References

[1] (2024). COPD chronic disease indicators. US Centers for Disease Control and Prevention.

[2] Weiss AJ, Jiang JH (2021). Overview of clinical conditions with frequent and costly hospital readmissions by payer, 2018, in HCUP statistical brief #278. https://hcup-us.ahrq.gov/reports/statbriefs/sb278-Conditions-Frequent-Readmissions-By-Payer-2018.jsp.

[3] Liu Y, Carlson SA, Watson KB, Xu F, Greenlund KJ (2023). Trends in the prevalence of chronic obstructive pulmonary disease among adults aged ≥18 years - United States, 2011-2021. MMWR Morb Mortal Wkly Rep.

[4] Mannino DM, Roberts MH, Mapel DW (2024). National and local direct medical cost burden of COPD in the United States from 2016 to 2019 and projections through 2029. Chest.

[5] Jencks SF, Williams MV, Coleman EA (2009). Rehospitalizations among patients in the Medicare fee-for-service program. N Engl J Med.

[6] Press VG, Myers LC, Feemster LC (2021). Preventing COPD readmissions under the hospital readmissions reduction program: how far have we come?. Chest.

[7] (2025). Defining the PCMH. US Department of Health and Human Services and Agency for Healthcare Research and Quality.

[8] Han MK, Martinez CH, Au DH (2016). Meeting the challenge of COPD care delivery in the USA: a multiprovider perspective. Lancet Respir Med.

[9] Doos L, Bradley E, Rushton CA, Satchithananda D, Davies SJ, Kadam UT (2015). Heart failure and chronic obstructive pulmonary disease multimorbidity at hospital discharge transition: a study of patient and carer experience. Health Expect.

[10] Lindenauer PK, Pekow P, Gao S, Crawford AS, Gutierrez B, Benjamin EM (2006). Quality of care for patients hospitalized for acute exacerbations of chronic obstructive pulmonary disease. Ann Intern Med.

[11] Health Quality Ontario (2017). Effect of early follow-up after hospital discharge on outcomes in patients with heart failure or chronic obstructive pulmonary disease: a systematic review. Ont Health Technol Assess Ser.

[12] Paasche-Orlow MK, Riekert KA, Bilderback A (2005). Tailored education may reduce health literacy disparities in asthma self-management. Am J Respir Crit Care Med.

[13] Press VG, Arora VM, Shah LM (2012). Teaching the use of respiratory inhalers to hospitalized patients with asthma or COPD: a randomized trial. J Gen Intern Med.

[14] Press VG, Arora VM, Shah LM (2011). Misuse of respiratory inhalers in hospitalized patients with asthma or COPD. J Gen Intern Med.

[15] Press VG, Arora VM, Kelly CA, Carey KA, White SR, Wan W (2020). Effectiveness of virtual vs in-person inhaler education for hospitalized patients with obstructive lung disease: a randomized clinical trial. JAMA Netw Open.

[16] Press VG, Arora VM, Trela KC (2016). Effectiveness of interventions to teach metered-dose and diskus inhaler techniques. A randomized trial. Ann Am Thorac Soc.

[17] Eisenhower C (2014). Impact of pharmacist-conducted medication reconciliation at discharge on readmissions of elderly patients with COPD. Ann Pharmacother.

[18] Mixon AS, Myers AP, Leak CL (2014). Characteristics associated with postdischarge medication errors. Mayo Clin Proc.

[19] Press VG, Au DH, Bourbeau J (2019). Reducing chronic obstructive pulmonary disease hospital readmissions. An official American Thoracic Society workshop report. Ann Am Thorac Soc.

[20] Locke ER, Thomas RM, Woo DM (2019). Using video telehealth to facilitate inhaler training in rural patients with obstructive lung disease. Telemed J E Health.

[21] Thomas RM, Locke ER, Woo DM (2017). Inhaler training delivered by internet-based home videoconferencing improves technique and quality of life. Respir Care.

[22] Emadi F, Dabliz R, Moles R (2025). Medication-focused telehealth interventions to reduce the hospital readmission rate: a systematic review. J Pharm Policy Pract.

[23] Thai T, Plotke M, Downing G, Olmsted E, Cook B, Jafri FN (2024). Telehealth pharmacist approach to comprehensive medication management in post-discharge high-risk patients: a quality improvement initiative. Telemed J E Health.

[24] Akula M, Nguyen M, Abraham J (2024). Determining if COPD self-management televisit-based interventions are evaluated among and equitably effective across diverse patient populations to reduce acute care use: a scoping review. Chest.

[25] Waltman A, Konetzka RT, Chia S (2023). Effectiveness of a bundled payments for care improvement program for chronic obstructive pulmonary disease. J Gen Intern Med.

[26] Abras C, Maloney-Krichmar D, Preece J (2004). Berkshire Encyclopedia of Human-Computer Interaction.

[27] Mao JY, Vredenburg K, Smith PW, Carey T (2005). The state of user-centered design practice. Commun ACM.

[28] Turner CW, Lewis JR, Nielsen J (2006). International Encyclopedia of Ergonomics and Human Factors.

[29] Bangor A, Kortum PT, Miller JT (2008). An empirical evaluation of the System Usability Scale. Int J Hum Comput Interact.

[30] Braun V, Clarke V (2006). Using thematic analysis in psychology. Qual Res Psychol.

[31] Fereday J, Muir-Cochrane E (2006). Demonstrating rigor using thematic analysis: a hybrid approach of inductive and deductive coding and theme development. Int J Qual Methods.

[32] Rojas Smith L (2014). Contextual Frameworks for Research on the Implementation of Complex System Interventions.

[33] Proudfoot K (2023). Inductive/deductive hybrid thematic analysis in mixed methods research. J Mix Methods Res.

[34] Bangor A, Kortum PT, Miller JT (2009). Determining what individual SUS scores mean: adding an adjective rating scale. J Usability Stud.

[35] Cypress BS (2017). Rigor or reliability and validity in qualitative research: perspectives, strategies, reconceptualization, and recommendations. Dimens Crit Care Nurs.

[36] Dransfield MT, Bailey WC (2006). COPD: racial disparities in susceptibility, treatment, and outcomes. Clin Chest Med.

[37] Shaya FT, Maneval MS, Gbarayor CM (2009). Burden of COPD, asthma, and concomitant COPD and asthma among adults: racial disparities in a medicaid population. Chest.

[38] Eisner MD, Blanc PD, Omachi TA (2011). Socioeconomic status, race and COPD health outcomes. J Epidemiol Community Health.

[39] Goto T, Faridi MK, Gibo K, Camargo CA, Hasegawa K (2017). Sex and racial/ethnic differences in the reason for 30-day readmission after COPD hospitalization. Respir Med.

[40] Nastars DR, Rojas JD, Ottenbacher KJ, Graham JE (2019). Race/ethnicity and 30-day readmission rates in Medicare beneficiaries with COPD. Respir Care.

[41] Fan VS, Gaziano JM, Lew R (2012). A comprehensive care management program to prevent chronic obstructive pulmonary disease hospitalizations: a randomized, controlled trial. Ann Intern Med.

[42] Griffiths S, Stephen G, Kiran T, Okrainec K (2021). “She knows me best”: a qualitative study of patient and caregiver views on the role of the primary care physician follow-up post-hospital discharge in individuals admitted with chronic obstructive pulmonary disease or congestive heart failure. BMC Fam Pract.

[43] Miravitlles M, Bhutani M, Hurst JR (2023). Implementing an evidence-based COPD hospital discharge protocol: a narrative review and expert recommendations. Adv Ther.

[44] Noel K, Messina C, Hou W, Schoenfeld E, Kelly G (2020). Tele-transitions of care (TTOC): a 12-month, randomized controlled trial evaluating the use of telehealth to achieve triple aim objectives. BMC Fam Pract.

[45] Rezende LC, Ribeiro EG, Parreiras LC (2023). Telehealth and telemedicine in the management of adult patients after hospitalization for COPD exacerbation: a scoping review. J Bras Pneumol.

[46] Rush KL, Hatt L, Janke R, Burton L, Ferrier M, Tetrault M (2018). The efficacy of telehealth delivered educational approaches for patients with chronic diseases: a systematic review. Patient Educ Couns.

[47] Le TV, Galperin H, Traube D (2023). The impact of digital competence on telehealth utilization. Health Policy Technol.

[48] Shaver J (2022). The state of telehealth before and after the COVID-19 pandemic. Prim Care.

[49] Sinha Gregory N, Shukla AP, Noel JJ (2023). The feasibility, acceptability, and usability of telehealth visits. Front Med.

[50] Fomo M, Borga LG, Abel T (2025). Empowering capabilities of people with chronic conditions supported by digital health technologies: scoping review. J Med Internet Res.

[51] Dantic DE (2014). A critical review of the effectiveness of ‘teach-back’ technique in teaching COPD patients self-management using respiratory inhalers. Health Educ J.

[52] O’Toole J, Krishnan M, Riekert K, Eakin MN (2022). Understanding barriers to and strategies for medication adherence in COPD: a qualitative study. BMC Pulm Med.

[53] Ramachandran HJ, Oh JL, Cheong YK (2023). Barriers and facilitators to the adoption of digital health interventions for COPD management: a scoping review. Heart Lung.

[54] Lennox L, Green S, Howe C, Musgrave H, Bell D, Elkin S (2014). Identifying the challenges and facilitators of implementing a COPD care bundle. BMJ Open Respir Res.

[55] Siu DCH, Gafni-Lachter L (2024). Addressing barriers to chronic obstructive pulmonary disease (COPD) care: three innovative evidence-based approaches: a review. Int J Chron Obstruct Pulmon Dis.

[56] Smith BJ, Dalziel K, McElroy HJ (2005). Barriers to success for an evidence-based guideline for chronic obstructive pulmonary disease. Chron Respir Dis.

[57] Bivolaru S, Constantin A, Vlase CM, Gutu C (2023). COPD patients’ behaviour when involved in the choice of inhaler device. Healthcare (Basel).

[58] Davis JR, Wu B, Kern DM (2017). Impact of nonadherence to inhaled corticosteroid/LABA therapy on COPD exacerbation rates and healthcare costs in a commercially insured US population. Am Health Drug Benefits.

[59] Baldwin-Medsker A, Holland J, Rodriguez ES (2020). Access to Care: using eHealth to limit location-based barriers for patients with cancer. Clin J Oncol Nurs.

[60] Galvin E, Desselle S, Gavin B, McNicholas F, Cullinan S, Hayden J (2024). Training service users in the use of telehealth: scoping review. J Med Internet Res.

[61] Lai AM, Kaufman DR, Starren J, Shea S (2009). Evaluation of a remote training approach for teaching seniors to use a telehealth system. Int J Med Inform.

[62] Ramadurai D, Lee CT, Traeger L (2025). Telehealth Education Leveraging Electronic Transitions Of Care for COPD Patients (TELE-TOC): a study protocol for a type II hybrid effectiveness-implementation randomised, pragmatic clinical trial of a pharmacist-led intervention. BMJ Open.

