# Supplementary material for: Patient-Centered Televisit for Chronic Obstructive Pulmonary Disease Discharge Transitions: User-Centered Design Study
Source: JMIR Hum Factors. 2025 Dec 5;12:e77953. doi: 10.2196/77953 (PMC12680292; doi:10.2196/77953)
Supplement: Multimedia Appendix 1 [file humanfactors-v12-e77953-s001.docx]

**APPENDIX**

**Table of Contents**

**S1.** **TELE-TOC Phase 1 Organization Leader Interview Guide**

**S2.** **TELE-TOC Phase 1 Clinician Interview Guide**

**S3.** **TELE-TOC Phase 1 Patient Interview Guide**

**S4. TELE-TOC Phase 1 Caregiver Interview Guide**

**S5. TELE-TOC Phase 2 Organization Leader Interview Guide**

**S6. TELE-TOC Phase 2 Clinician Interview Guide**

**S7. TELE-TOC Phase 2 Patient Interview Guide**

**S8. TELE-TOC Phase 3 Clinician Interview Guide**

**S9. TELE-TOC Phase 3 Patient Interview Guide**

**S1.** **TELE-TOC Phase 1 Organizational Leader Interview Guide**

**Baseline Questions**

1. How would you describe your role and responsibilities as an organizational leader?
2. In what ways does your role allow you to address COPD patient care needs during hospitalization, at discharges, and with outpatient follow-up care?

**Intervention Technology skills/needs/comfort:** We hope to design a clinic visit that can happen via video while a patient is at home.

1. Do you think at-home tele-visits for medication review and reconciliation would be of value?
   1. If yes- in what way
   2. If no- why
2. Do you think at-home tele-visits for COPD mediation education and training would be of value?
   1. If yes- in what way
   2. If no- why
3. Have you had a role in helping with the implementation or design of other tele-visits or technology-based clinic visits?
   1. If yes, how did you help with the design?
   2. What were some needs of the hospital and/or clinic that needed to be addressed?
   3. What were some accomplishments/improvements you noticed with the video visits vs in-person clinic visits?
   4. How did you ensure usability and feasibility for both the person conducting the video visit and the patient?
4. What would be some of the barriers to telehealth (video) visits for COPD management including medication reconciliation and/or patient COPD education (e.g., medications):
   1. What would be needed to overcome these barriers?
   2. Are there any equity concerns (prompts- e.g., age, SES, digital literacy, tech access)
   3. If you’ve had any experience with video visits at all, what are some lessons learned you can share?
5. What resources would be needed to ensure a successful telehealth video visit program for COPD medication management including reconciliation and education?
   1. Who would help obtain these resources
6. Is there anything you can think of to make the experience better for a patient and clinician?
7. Before we conclude this session, we have one last question that covers all that we discussed today: If you had to share any lessons learned, tips, and information (that you had previously wished for yourself and/or for a patients) to help patients with COPD or anyone involved in the care of patients with COPD, what would you include?

**At Hospital Discharge**

About hospital discharge (We are interested in learning more about the process at the end of hospitalization for patients to help them get discharged from the hospital and ready to resume managing their COPD at home).

1. Please describe your understanding of the processes that are in place for patients during hospitalization and at hospital discharge.

*Medication Reconciliation for administrators or organizational leaders*

1. While patients are hospitalized, what is the process for ensuring successful medication reconciliation? Is this process the same or different for patients with COPD? Any hospital metrics you think are relevant to this that you track at the hospital level?
   1. How in your role could you impact this?
2. What metrics are used to determine the success of the medication reconciliation portion of COPD care?
3. We are interested in learning more about how to make the medication management in the hospital more useful. What were some of the barriers you see with medication management or when developing a program to improve this process?
4. What methods, processes, resources help with regards to medication management? Please explain if there is anything that you would keep in the current hospital discharge process.
5. Please explain if you would suggest anything different in this process. Anything that could help leaders like you to understand, manage, and develop a program to this.

*COPD Education for administrators or organizational leaders*

1. While a patient is hospitalized, what is/could be your role in developing and/or ensuring the success of COPD education delivery?
   1. What metrics are used to determine the success of the COPD education?
2. Please explain why you do or do not think current COPD education during hospitalization is successful/beneficial to patients.
3. What is your knowledge of COPD education provided to patients?
   1. If education provided…at what part of the patient’s admission is education conducted? Please describe what you feel the education entails.
   2. If education is not provided, do you feel like it should be?
4. What are/were barriers to delivering COPD education or challenges that could exist when developing COPD education programs?
5. What works well currently with regards to COPD education? Please explain if there is anything that you would keep in the current process during hospitalization and discharge.
6. Please explain if you would suggest anything different in this process. Are there any resources you could think of that could help leaders like you to understand, manage, and develop COPD health education programs?

**At Home Care**

Once a patient is home, we want to know what your role is as it relates to their care

1. What is your understanding of what is available for at-home medication review/reconciliation?
2. What resources should be available? Do you think there are opportunities for improvement?
3. Do you believe having at-home med rec/review options could help avoid revisits to ED or hospital?

**Outpatient Follow-up**

*Medication Reconciliation for administrators or organizational leaders.*

1. What metrics are used to determine success of medication review during COPD outpatient visits?
2. Please explain why you do or do not think the medication review portion of care is beneficial to the patient
3. Do clinicians review medications with their patients during clinic visits?
   1. If yes, are you familiar with the medication process during a clinical visit?
   2. If yes, who is the clinician doing this review?
   3. If yes, what part of the patient’s visit did the medication review occur?
   4. If yes, please describe what you believe the medication review entails.
   5. If no, do you think medication reconciliation should occur in the clinic visit?
   6. If no, who is conducting medication review? Do you believe clinicians should be conducting the review?
4. What are some of the barriers that exist for outpatient medication reconciliation (prompts: developing/implementing processes, developing/obtaining metrics for outpatient medication reconciliation)?
5. What do you think currently works well about this process? Please explain if there is anything that you would keep in the current outpatient process.
6. Please explain if you would suggest anything different in this process. Anything that could help leaders like you to understand, manage, and facilitate development of a medication reconciliation program.

*COPD Education for administrators or organizational leaders*

Note to interviewer: this is an open-ended question.

1. What metrics are used to determine success of COPD education delivery care during outpatient clinical visits?
2. Please explain why you do or do not think the education portion of care is beneficial to the patient
3. Does COPD education occur in outpatient visits?

*Note to interviewer: this is a yes or no question with follow up questions. prompts: in primary care, pulm specialty visits*

- 1. If yes, what part of the patient’s visit did the COPD education occur? Please describe what you think the COPD education entails.
  2. If no, do you think education should be provided?

1. What are some of barriers for delivering COPD education during outpatient visits?
2. What do you think currently works well about this process? Please explain if there is anything that you would keep in the current outpatient process.
3. Please explain if you would have preferred anything different in this process. Anything that could help leaders like you to understand, manage, and develop a COPD education program similar to this.

**S2. TELE-TOC Phase 1 Clinician Interview Guide**

**Baseline Questions**

1. Please describe your role as it relates to patient care delivery
   1. How are you involved in patient care in clinical settings (inpatient, outpatient)? What are your clinical roles/ responsibilities?
   2. Do you have experience working with patients with COPD?
      1. If yes, how long have you worked with patients with COPD?
      2. If yes, how many patients with COPD do you work with on average each month?
      3. Could you please describe your interactions with patients with COPD in the inpatient or outpatient setting?

**At Hospital Discharge**

1. Explain your role and involvement in the discharge process.
2. Please describe the clinical workflow related to the discharge process and your role in that process.

*Medication Reconciliation for physicians, nurses, pharmacists, advanced practitioner providers or respiratory therapists, community health workers or social workers*

1. During a patient’s hospitalization, do you review their medications?
   1. If no/maybe, please can you describe the answer a little.
   2. Who, if anyone, reviews their medication?
      1. When does this person review their medications?
      2. How do they make sure they are thorough with medication review/reconciliation?
      3. How do they confirm with a patient/caregiver that they fully understand their medications and instructions?
      4. How do they make sure they are thorough with medication review/reconciliation?
      5. How do they confirm with a patient/caregiver that they fully understand their medications and instructions?
   3. Can you describe at what point or when do you review a patient’s medications?
   4. Who else might review their medications?
2. How good do you think the quality of the medication reconciliation is for patients? Likert: 1) very low quality; 2) low quality; 3) neutral; 4) high quality; 5) very high quality
   1. Tell us a little more about what you mean by your answer “1-5”.
   2. Do you think your answer would change for low-risk patients vs. high-risk patients? How?
   3. Do you think your answer would change for low-literacy patients vs. high-literacy patients? How?
   4. How would you describe “high quality” medication reconciliation?
   5. How would you describe “low quality” medication reconciliation?
3. How prepared do you think patients (and/or their caregivers) are after you have reviewed their medications? (i.e., med rec) Likert: 1) not at all prepared; 2) slightly prepared; 3) somewhat prepared; 4) very prepared; 5) extremely prepared.
   1. Tell us a little more about what you mean by your answer “1-5”.
   2. Would your answer change if we were asking about high-risk or low literacy patients? How?
4. In your role as a [clinician type], how do you make sure you are thorough with medication review/reconciliation?
5. How do you confirm with a patient (and/or their caregiver) that they fully understand their medications and related instructions?
6. What are some of the barriers that patients typically experience related to medication management?
7. What are some of the barriers you face related to medication management?
8. What impact does the “insert barrier” have on taking care of their COPD?
9. What do you typically provide that works well with regards to medication management? Please explain if there is anything that you would retain in the current hospital discharge process.
10. Please explain if you would suggest anything different in this process. Anything that could help clinicians like you to understand and manage patient’s medications.

*COPD Education for physicians, nurses, pharmacists, advanced practitioner providers, or respiratory therapists, community health workers or social workers*

1. During a patients’ hospitalization, do you provide any information or educate them about their COPD care management and/or their medications?
   1. If no, please explain why not? Do you wish you did?
   2. Does anyone else provide information or COPD education?
      1. If so, who?
      2. When do they provide this information or education?
2. What information or education do you wish you or someone else could’ve provided?
   1. If yes/maybe, can you please explain what information/education you provide?
   2. When, during the hospitalization, do you typically provide this information?
   3. Tell us more about the experience of providing this information/education about COPD during their hospitalization
3. How do you make sure you are thorough in providing education?
4. If you do review the patient’s medications, how do you ensure a patient understands how to use their medications? Do you do provide inhaler technique education or information?
   1. If yes, please describe your answer further/what this information or education looks like.
5. What are some of the barriers the patient experiences related to education?
6. What are some of the barriers you faced related to education?
7. What impact does the “insert barrier” have on taking care of their COPD?
8. What do you typically provide that works well with regards to providing education? Please explain if there is anything that you would keep in the current hospital discharge process.
9. Please explain if you would suggest anything different in this process.
10. Anything that could help clinicians like you to educate patients with COPD on their care management.

**At Home Care**

1. Explain your role and involvement in home care management after the patient is discharged from the hospital.

*Medication review and reconciliation for physicians, nurses, pharmacists, advanced practitioner providers, or respiratory therapists, community health workers or social workers*

1. Once a patient goes home, do you check to make sure a patient has refilled all their medications and/or provide any tools/resources related to COPD education? Why or why not?
   1. If no, does anyone else check on the patient’s medication fills or follow-up with tools/resources?
      1. If yes, who?
   2. If yes, how do you assess if the patient understands which medications to take?
   3. If yes, how often do you contact patients about these topics?
   4. What kind of tools and resources do you provide?
2. At home, how do you assess if the patient understood which medications to take?
3. Do you have any role when it comes to patient’s continued medication management at home? Given your role with home care, what are some of the common barriers patients experience related to medication management at home?
4. What impact does the “insert barrier” have on taking care of their COPD?
5. What are some of the common barriers you face related to medication review and management at home?
6. What do you provide that works well with regards to medication management at home? Please explain if there is anything that you would keep or currently do to enhance a patient’s home care.
7. Please explain if you would suggest anything different in this process. Anything that could help folks like you to understand and manage their patient’s home care.

**Outpatient Follow-up**

1. Explain your role as it relates to a patient’s follow up appointment.
2. Who schedules and/or when does a patient’s follow-up appointment get scheduled?
   1. If you, do you usually schedule during their hospitalization, when they are being discharged, once the patient’s been home, etc.?
   2. How do you ask a patient to prepare for their follow up? Do you ask them to bring their medications, explain what will happen during the appointment, etc.
   3. If not you, do you know who does schedule the appointment? When do they schedule?
3. What does the follow-up appointment with a patient look like?
   1. Please walk us through a typical appointment from start to end.
4. If you are not present at the appointment, do you feel like you need to be, do you have any communication with the patient before or after their appointment, etc.?
   1. Do you know who is present at the appointment?

*Medication review and reconciliation for physicians, nurses, pharmacists, advanced practitioner providers, or respiratory therapists, community health workers or social workers*

1. At the follow-up visit, do you review the patient’s medications?
   1. If no, does anyone else review the patient’s medications?
   2. If yes, who?
      1. When does this person review their medications?
      2. How do they assess if the patient understands which medications to take?
      3. How do they make sure they are thorough with medication review/reconciliation?Can you describe at what point or when do you review a patient’s medications?
         1. Who else might review their medications?
         2. How do they make sure you are thorough with medication review/reconciliation?
         3. How do you assess if the patient understands which medications to take?
2. Any issues you encounter during the clinic visits of patients with COPD?
3. What are some of the common barriers you face related to medication review and management at follow-up visits?
4. What aspects of the follow-up clinic appointment do you feel help your role to better manage their care? Please explain.
   1. What do you provide that works well with regards to medication management at follow-up? Please explain if there is anything that you would keep or currently do to enhance a patient’s follow-upcare.
5. What should be done differently during the clinic visit to better help you, another provider, or a patient with their medications?
   1. Would support and expertise from a tele-health COPD support team be appreciated and help address some of the barriers you mentioned?
      1. If no, could you explain why not?

*COPD Education for physicians, nurses, pharmacists, advanced practitioner provider, or respiratory therapy, community health worker or social worker*

1. During follow-up, do you provide any information or educate patient’s about their COPD care management and/or their medications?
   1. If no:
      1. Please explain why not? Do you wish you did?
      2. Does anyone else provide information or COPD education?
         1. If so, who?
         2. When do they provide this information or education?
   2. If yes/maybe, can you please explain what information/education you provide?
      1. When during the appointment do you typically provide this information?
      2. Tell us more about the experience of providing this information/education about COPD during the follow-up
2. If you are not present at the appointment, do you feel like you need to, do you have any communication with the patient before or after their appointment, etc.?
3. Any additional barriers/challenges with regards to COPD education at the time of follow-up visit?
4. Any facilitators that you or other clinicians use often to enable the COPD education at the time of follow-up visit?
5. What should have been done differently during the clinic visit to better help you/ a patient with COPD education?
   1. Would support and expertise from a tele-health COPD support team be appreciated and help address some of the barriers you mentioned?
      1. If no, could you explain why not?

**Intervention Technology Skills/needs/comfort**

1. Please describe what your experience has been like conducting a clinical visit over video.
   1. If you have a done a clinic visit over video,
      1. What are some barriers you face when conducting them?
      2. How do you conduct them? What are some topics you go over? Is it similar to how you conduct them in-person?
      3. If it was for COPD or for post-hospital discharge:
      4. How do you ensure a patient understands the information being shared with them?
         - 1. What some barriers do you think patients experience?
      5. What are some triumphs and successes you can share to help other hospitals and clinics utilize video visits?
      6. Would you be willing to assist them with a video visit to learn more about your medications/ in general about COPD management at home? How so?
   2. you have not done a clinic visit over video,
      1. Why not?
      2. What would encourage you to start conducting video visits?
2. Would you be willing to assist a patient with a video visit to learn more about your medications/ in general about COPD management at home? How so?

**S3. TELE-TOC Phase 1 Patient Interview Guide**

**Patient Discharge Transition Flow (Going Home from the Hospital)**

*Before asking you some questions, take this piece of paper and write down or draw a picture of all the things that the doctors and nurses did or talked to you about in the hospital when you were getting ready to go home.*

*Notes to interviewer:*

*Think about the things that you have to do or things that happen for your COPD when you are being discharged from the hospital and then once you get home. Some example prompts are:*

- *To take care of your COPD*
- *To talk to your doctor or nurse about your COPD*
- *To get your medications*
- *To make an appointment for your follow-up visit for your COPD*

1. What things helped you? Why do these help you?
2. How do each of these things help you take care of your COPD?
3. Are there things that do not help you? Why are these not helpful?
4. For each of the things that don’t help you, how can things be done better to help you take care of your COPD

**At Hospital Discharge**

*Now we are going to ask some specific questions. First, we want to talk about your care when you were in the hospital getting ready to go home. We want to know about Medication Review.*

1. When you were still in the hospital, did anyone talk with you or “go over” your medications at any time?
2. Who talked with you?
3. When did [insert person] review your medications with you?
4. Did anyone talk with you about what medications you needed to take at home?
   1. If YES: When did [insert person] review your medications with you?
   2. If YES: Did they talk to you on the day you were going home?
   3. If YES: Was anyone else (i.e. husband, wife, daughter, friend, other clinician, etc.) with you at this time?
   4. If YES: Can you tell me what the [doctor, pharmacist, nurse] told you about your medications?
   5. If YES: Did they review your current list of all your medications (old, new, stopped meds)?
      1. If YES: What did the [doctor, nurse, pharmacist, etc.] go over with you specifically?
      2. If YES: Did the [doctor, nurse, pharmacist, etc.] talk about any new medications that were prescribed to you while you were in the hospital?
   6. If YES: Did the [doctor, nurse, pharmacist, etc.] review medications that were stopped/discontinued?
      1. If YES: Did the [doctor, nurse, pharmacist, etc.] talk about reasons for adding/discontinuing these medications?
      2. If YES: Did the [doctor, nurse, pharmacist, etc.] give you an updated list of medications before you were sent home?
         1. If YES: When was it provided?
   7. If NO: Would you have wanted someone to talk with you about this?
      1. If NO: What information about your medications would you have wanted to review?
      2. If NO: When during your stay in the hospital would you have wanted the [doctor, nurse, pharmacist, etc.] to review your medications with you?
      3. If NO: Who would you want to review your medications with you?
5. Were your new or current medications filled and given to you before you left the hospital?
6. Tell me from 1 to 5 with 1 being “NOT prepared” and 5 being “EXTREMELY prepared”, how “prepared” did you feel about taking your medications when you returned home?
   1. Tell me a little more about what you mean by your answer.
      1. Did you know what you were supposed to take?
      2. Did you know when to take them?
      3. Did you know how to take them?
      4. Did you understand the instructions and the reason for taking each medication?
      5. Did you read the paper or “insert” that comes with each medication?
7. What was provided that worked well about medication review while you were in the hospital?
   - 1. What things would you tell your [doctor, nurse, pharmacist] to keep about reviewing medications during the hospital stay?
8. What can we do to help patients like you better understand how to take your medications?

*Now we are going to ask you some questions about whether anyone talked to you about COPD Education while you were in the hospital*

1. While you were in the hospital, did anyone give you any information or teach you about your COPD?
   1. If YES/MAYBE: What information or teaching about COPD was provided to you?
   2. If YES/MAYBE: Did you find the information/teaching helpful?
   3. If YES/MAYBE: Was there information/teaching you wished you had gotten?
   4. If YES/MAYBE: When did you receive this information?
   5. If YES/MAYBE: Was the timing ok?
   6. If YES/MAYBE: Would you have wanted information/teaching earlier or later in your hospital stay?
   7. If YES/MAYBE: Were you able to understand the information/education provided to you?
   8. If YES/MAYBE: What information did you feel like was missing or needed from education that could have helped you?
   9. If YES/MAYBE: Was the COPD education thorough? Did the information/education provided help you take care of your COPD during your hospital stay?
   10. If YES/MAYBE: Would you have liked to get this information/teaching after you left the hospital?
2. Did you get information/education about your medications while you were in the hospital?
   1. If YES/MAYBE: Did you find the information/education helpful?
   2. If YES/MAYBE: Was there information/education you wished you received?
   3. If YES/MAYBE: Was the timing ok?
   4. If YES/MAYBE: Would you have preferred it earlier/later in the hospital stay? Or not during the hospital stay?
   5. If YES/MAYBE: Were you able to use the education/information to help you take your COPD medications?
   6. If NO for either or both COPD education and medication education:
      1. What information or education would you want to receive?
      2. When during the hospitalization would you have wanted the information?
      3. Would you want COPD information and education also outside of your hospitalization?

*We want to help patients learn better about their medications while they are still in the hospital.*

1. What were some problems you experienced in the hospital related to learning about your medications or about your COPD care?
   1. How did the problems you experienced impact your COPD care while you were in the hospital?
   2. While the [doctor, nurse, pharmacist, etc.] was reviewing your medications and/or COPD education, what information did you feel like was missing or needed?
   3. Did you notice any mistakes in the medication list or the information that they gave you?
      - 1. If YES: Can you tell me what mistake(s) you noticed?
   4. Did you feel that the [doctor, nurse, pharmacist, etc.] went over your medications completely? Did you feel that the education was complete?
   5. Did you have any questions for the [doctor, nurse, pharmacist, etc.] that were not answered?
      1. If YES: What questions?
2. Please explain if you would have preferred anything different in this process. Anything that could help patients like you to understand and learn better.

**At Home Care**

1. After go home from the hospital, we want to know how things went for you when taking care of your COPD.
2. Were you able to get all your COPD medications from the pharmacy or hospital?
3. Did you understand which medications to take?
   1. If not, why?
4. Were there any medications you didn't take or stopped taking?
   1. If yes, why?
   2. If patient DID NOT receive COPD education in the hospital:
      1. Would it have helped you at home if you had gotten some information about COPD or teaching about your medications in the hospital?
   3. Would instructions and teaching on how to use your inhaler(s) have helped you at home?
5. What helped you with taking your medications after you got home?
6. If patient DID receive education in the hospital:
   1. From 1 to 5, where 1 means strongly disagree and 5 means strongly agree, how much do you agree with this statement: “The information I got in the hospital helped me know when and how to take my medications.”
      1. Tell us more about what you mean by your answer
   2. Now, from 1 to 5, where 1 means strongly disagree and 5 means strongly agree, how much do you agree with this statement: “The instructions on how to use the inhaler(s) helped me.”
      1. Tell us more about what you mean by your answer
7. What problems did you have with your medications at home?
8. What problems did you have taking care of your COPD care once you got home?
   1. If on oxygen: Issues with oxygen? Wasn't delivered, not portable, other?
   2. Felt stuck at home or hard to go out?
      1. If YES: why? Oxygen, breathing, etc.?
9. What changed for you, if anything, after you got home from the hospital in terms of taking care of your COPD?
10. What stayed the same for you after you got home from the hospital in terms of taking care of your COPD?
11. What questions did you have for the [doctor, nurse, pharmacist, other] after you got home?
12. Did anyone from your hospital stay call to check on you after discharge?
    1. Did you have a way to get in touch with them?
    2. Did you have someone else to call? Primary care clinician? Lung clinician?

**Outpatient Follow-up**

*After you were sent home from the hospital, we want to know how things went if you were seen in a clinic.*

1. Did you have a clinic appointment scheduled for after your hospital stay?
2. Note to interviewer: this is a yes/no question with open-ended questions to follow up with.
   1. If YES: Did you get the date and time of your appointment before you left the hospital?
   2. If YES: Who was the appointment with?
   3. If NO: Do you wish an appointment had been made?
   4. If NO: Which doctor/nurse would you have wanted an appointment with?
   5. If NO: What would you have wanted to talk about at this follow-up visit?
3. Were you able to go to your follow-up visit?
   1. If NO: Why did you not make your appointment?
   2. If NO: What kinds of things stopped you from being able to make your follow-up visit?
   3. If NO: Would a telehealth visit have helped you make your clinic visit?
   4. If YES: How did you get ready for your follow-up appointment?
   5. If YES: Did you bring your medications or a list of medications to the appointment?
   6. If YES: Tell me about your clinic appointment. What did the [doctor, nurse, pharmacist etc.] talk about with you during the visits?
   7. If YES: Did the [doctor, nurse, pharmacist etc.] give you a checklist, printed documents, brochure, website or anything else to help you keep track of what was explained or to help you take care of your COPD?
   8. If YES: Did the [doctor, nurse, pharmacist etc.] give you a phone number you can call if you need help?
   9. If YES: How long was your appointment?
   10. If YES: Did someone review your medications with you during the clinic visit?
       1. If YES: Who went over your medications with you?
       2. If YES: What did they go over?
       3. If YES: Did they ask you about any problems with your medications or any new symptoms?
       4. If YES: Were you given information or taught about your medications?
          1. If YES: Were you able to understand these instructions?
       5. If NO: Do you wish someone would have gone over things with you?
          1. If YES: Why?
          2. If NO: Why not?
   11. If YES: Did anyone show you how to use your inhaler(s)?
   12. If YES: Were changes made to your medications?
       - 1. If YES: Were the reasons for the changes explained to you?
   13. If YES: What else was discussed during the visit?
   14. If YES: Was there any new information or education given to you during the clinic visit compared to when you were in the hospital?
       1. If YES: how was it different than what you got in the hospital?
   15. If YES: What other things about care of your COPD did [doctor, nurse, etc.] and you decide on together?
4. Did you schedule any other clinic appointments with a doctor, nurse, pharmacist, specialist, etc.?
5. Tell me from 1 to 5 with 1 being “NOT prepared” and 5 being “EXTREMELY prepared”, how “prepared” did you feel about caring for your COPD after your follow-up appointment?
6. Note to interviewer: Use the Likert scale to confirm their answer, and then ask the probing follow-up questions.
7. What problems did you have during the clinic visit, if any?
   1. Did you have problems with the medication review during your clinic visit?
      1. If YES; What were they?
8. Note to the interviewer: this and the following are open-ended follow up questions. Probes: issues with refilling medications, insurance coverage issues, not knowing when to take the medications, not knowing how to take the medications.
   1. Did you have problems with the COPD education during your clinic visit?
      1. If YES; What were they?
9. Were you able to understand the information you received?
   1. If NO: What was difficult to understand?
      1. While the [doctor, nurse, pharmacist] was providing COPD information or teaching, what information did you feel like was missing?
      2. Did you have any questions for the [doctor, nurse, pharmacist] that were not answered?
         - 1. If YES: What were they?
      3. Did you notice any mistakes in the information/teaching given to you? Please describe.
10. What worked well and why?
    1. How did this information change how you care for your COPD?
    2. What parts of the clinic appointment helped you better take care of your COPD?
    3. Were you able to use the education/information you to take care of your COPD?
    4. Was there anything that could have helped you to understand the information or teaching better.
11. Is there anything that could have been done differently during the clinic visit to help you with your medications or to take care of your COPD?

**Intervention Technology skills/needs/comfort**

*We hope to design a telehealth clinic visit that can happen over video to help with taking your medications for COPD. We call health visits that happen over video “telehealth” visits.*

1. Have you had a video telehealth visit with a doctor or nurse?
   1. If NO: What has kept you from having a video visit?
   2. If NO: Is there anything that would help you with health-related video visits?
   3. If YES: Can you describe your telehealth visit?
   4. If YES: What happened?
   5. If YES: What was the visit for?
   6. If YES: How did you like the experience?
   7. If YES: How does it compare to an in-person visit?
   8. If YES: What did you miss out from an in-person visit, if anything?
   9. If YES: Was it for your COPD or after discharge from the hospital?
   10. If YES: Which [doctor, nurse, pharmacist, etc.] did you see during the telehealth visit?
   11. If YES: Did they review your medication list during the telehealth visit?
   12. If YES: Did they show you how to use your inhaler(s) during the telehealth visit?
   13. If YES: Is there anything that can be done to make the experience better for you?
2. Would you be interested in having a telehealth video visit to learn more about your medications and how to take care of your COPD at home?
3. If you had to share any lessons learned, tips, and information (that you had previously wished for yourself) to help caregivers of patients with COPD, what would you include?

**S4. TELE-TOC Phase 1 Caregiver Interview Guide**

**Baseline Questions**

1. How are you related to [name of person with COPD]?
2. How long have you been taking care of this individual?
3. Do you work outside the home?
4. Did you work outside the home before you started caring for [insert patient’s name]?
5. How much time do you spend taking care of [insert patient’s name] COPD and medications?
6. In your words, how would you describe what you do for [insert patient’s name] to help them with their COPD?
   1. What tasks do you help with?
7. Did you have any training or education in how to care for a patient with COPD?
   1. If YES: Where did you get this training or education?
8. Probes: In the hospital before [name of person with COPD] was sent home, in the clinic, in the Emergency Room, through an official training or licensing program, etc.
   1. If YES: Who talked to or taught you?
   2. If YES: What did the training include or what was talked about?
   3. If YES: Did you feel that the training or teaching helps you take better care of [insert patients’ name]?
   4. If YES: Did you feel the training helped you better understanding how to care for patients with COPD and what can happen with their illness?
   5. If NO: Do you wish that you had training or education?
      1. If YES: For COPD care specifically?
      2. If YES: What do you think someone should have taught you or told you that would help you care for patients with COPD?
9. How dependent is [insert patients’ name] on you for helping with their COPD care?
10. How has taking care of [insert patients’ name] affected or changed your personal life? Please elaborate on how your personal life has been impacted.
11. Is taking care of [insert patients’ name] stressful or hard for you?
    1. If YES: what sorts of things make it stressful or hard for you?

*A tele-health visit is a visit with a doctor/nurse/or pharmacist over the internet. The patient uses his/her camera and microphone on a smart phone, an ipad, or a computer to meet with the doctor/nurse/or pharmacist. Of course, the doctor/nurse/or pharmacist can’t examine the patient but can look at and talk to him/her.*

1. Have you ever had a tele-health visit yourself or has [name of person with COPD] ever had a tele-health visit?
2. Do you think a tele-health visit with a doctor/nurse/pharmacist who cares for patients with COPD be helpful for you?
   1. Why or Why not?
   2. If NO: Do you have any other help to make it less stressful or hard to take care of [name of person with COPD]?
   3. If NO: Do you think a tele-health visit with a doctor/nurse/pharmacist who cares for patients with COPD be helpful for you?

**At Hospital Discharge**

*Now, I am going to ask you some questions about your experience in the hospital when [name of person with COPD] was getting ready to be sent home Explain your role and involvement in the discharge process.*

1. How much were you involved or take part in getting [name of person with COPD] ready to go home from the hospital?
2. Were you asked to be at the hospital when the doctor/nurse were getting [name of person with COPD] ready to go home?
3. While [insert patients’ name] was in the hospital, did anyone talk with you about their medications or what they should be taking when they went home?
   1. If YES: who talked with you?
   2. If YES: When did they talk to you?
   3. If YES: Who else was present at this time besides you and the patient?
   4. If YES: What was reviewed and discussed with you and the patient?
   5. If YES: Did they review your current list of all the patient’s medications (old, new, stopped meds)?
      1. If YES: What did the [doctor, nurse, pharmacist, etc.] go over specifically?
      2. If YES: Did the [doctor, nurse, pharmacist, etc.] talk about any new medications that were prescribed to the patient while in the hospital?
   6. If YES: Did the [doctor, nurse, pharmacist, etc.] review medications that were stopped/discontinued?
      1. If YES: Did the [doctor, nurse, pharmacist, etc.] tell you why a medication was stopped?
      2. If YES: Did the [doctor, nurse, pharmacist, etc.] give you an updated list of medications for the patient before they were sent home?
         1. If YES: When was it provided?
   7. If NO: Would you have wanted someone to talk with you about the patient’s medications while they were in the hospital?
   8. If NO: What information about the patient’s medications would you have wanted to review?
   9. If NO: When during the patient’s hospital stay would you have wanted the [doctor, nurse, pharmacist, etc.] to review the patients’ medications with you?
   10. If NO: Who would you want to review the medications with you?
4. How prepared did you feel to take care of [name of person with COPD] after someone went over [name of person with COPD]’s medications? 1= not at all prepared; 2= slightly prepared; 3= somewhat prepared; 4= very prepared; 5= extremely prepared.
   1. Tell us a little more about what you mean by your answer “1-5”
5. Did you feel like you knew [insert patients’ name] was supposed to take?
   1. When to take?
   2. How to take?

*Now, I am going to ask you some questions about what would make it easier for you to take care of [name of person with COPD].*

1. What were some of the problems or issues that you and [name of person with COPD] have had with [name of person with COPD]’s medications?
2. When someone was going over [name of person with COPD]’s medications with you, did you feel like some information was missing or more information was needed?
3. Did you have any questions for the [doctor/nurse/pharmacist] that were not answered?
   1. If YES: What were they?
4. Were you able to understand the instructions for each medication and the reason for taking each medication?
   1. If NO: How did NOT understanding the instructions make it harder for you to take care of [name of person with COPD]?
5. What worked well for you with the medication instructions?
6. What things worked well or did the [doctor/nurse/pharmacist] do while they were getting ready for the patient to go home from the hospital that helped the patient or helped you care for the patient?
7. Would you have preferred anything to have been done differently when you were getting [name of person with COPD] ready to go home that could help caregivers like you to understand and manage a patient’s medications better.
8. During [name of person with COPD]’s stay in the hospital, did anyone talk to or teach you about COPD in general or provide inhaler teaching to you or the patient?
   1. If YES: What information were you provided or what were you taught?
   2. If YES: When during the hospital stay did someone talk or teach you about COPD?
   3. If YES: What worked well?
   4. If YES: Were you able to use what you were told or taught after [name of person with COPD] got home from the hospital?
   5. If YES: Were you told or taught everything that you needed to know/learn about COPD?
   6. If YES: Did you understand what you were told or taught about COPD?
   7. If YES: Did you notice any errors/mistakes that were told or taught to you?
      1. If YES: What errors or mistakes did you notice?
   8. If YES: Did you have any questions for the doctor, nurse, pharmacist that were unanswered?
      1. If YES: What were they?
   9. If NO: Do you wish that someone had talked to or taught you as [name of person with COPD]’s caregiver about COPD?
   10. If NO: What would you have wanted to be told or taught?
   11. If NO: When during [name of person with COPD]’s stay in the hospital would you have wanted someone to talk or teach you about COPD?
   12. If NO: Would you want someone to talk to or teach you about COPD outside of [name of person with COPD]’s stay in the hospital?
9. Would you have wanted any different COPD education or information during the time when the patient was going from the hospital back home that could help caregivers like you care for patients with COPD better?

**At Home Care**

*Now, I’d like to ask you a few questions about taking care of [name of person with COPD] at home.*

1. Explain your role and involvement in home care.
2. Were you able to fill all the medications that [name of person with COPD] needed from the pharmacy or hospital?
3. Did you feel that [name of person with COPD] understood which medications to take?
   1. If NO: why?
4. Were there any medications they didn't take/stopped taking?
   1. If YES: why?

*If patient/caregiver DID receive education in the hospital:*

1. How much do you agree with this statement on a scale of 1-5: The information and/ or teaching that I got in the hospital helped me know when and how to provide medications to [name of person with COPD]. Likert scale: Strongly Agree, Agree, Neutral, Disagree, Strongly Disagree
   1. Tell us a little more about what you mean by your answer “1-5”
2. How much do you agree with this statement on a scale of 1 -5: The instructions on how to use the inhaler enabled me to help the patient with their inhaler. Likert scale: Strongly Agree, Agree, Neutral, Disagree, Strongly Disagree
   1. Tell us a little more about what you mean by your answer “1-5”

*If caregiver did NOT receive COPD education or information in the hospital:*

1. Would instructions on how to use [name of person with COPD]’s inhalers would have helped?
2. What did you as a caregiver struggle with the most when giving [name of person with COPD] his/her medications?
3. What helped your care recipient with taking their medications when you got home?
4. What challenges existed for [name of person with COPD] after he/she got home? Felt stuck at home or hard to go out?
5. What are some things you did differently when caring for [name of person with COPD] after he/she got home?
6. What are some things that stayed the same for you when taking care of [name of person with COPD] after he/she got home?
7. What questions did you have for the doctor, nurse, or pharmacist after [name of person with COPD] got home?
   1. Did you know how to get in touch with the clinicians when you had questions?
   2. Were you able to get your questions answered?
      1. If NO: why not?

**Outpatient Follow-up**

1. *Now, I am going to ask you some questions about any clinic visits that [name of person with COPD] had after going home from the hospital.*
2. After [name of person with COPD] got home, was he/she seen in clinic?
   1. If YES: Was the clinic appointment scheduled for [name of person with COPD] before he/she left the hospital?
   2. If NO: Did you wish that a clinic appointment had been scheduled for [name of person with COPD] before he/she was sent home from the hospital?
   3. If NO: Did you have trouble trying to make a clinic appointment for [name of person with COPD] after he/she got home from the hospital?
3. Would a tele-visit work for your care recipient for this purpose? Would this have been convenient for you to join this tele-visit? Please elaborate on it.
4. Were you able to go to the follow-up clinic appointments?
   1. If YES: How did you and [name of person with COPD] get ready for the clinic visit?
   2. If YES: Did [name of person with COPD] bring their medications or a list of medications to the appointment?
   3. If YES: Can you describe the clinic appointment? What did the [doctor/nurse/pharmacist] talked with you and the patient about related to COPD?
   4. If YES: Did the doctor/nurse/pharmacist give you any things like a brochure, a website, a checklist, an action plan to help you as [name of person with COPD]’s caregiver?
   5. If YES: What did the doctor/nurse/pharmacist give you?
   6. If YES: Did the doctor/nurse/pharmacist give you a phone number that you and [name of person with COPD] can call if you had questions or concerns?
   7. If YES: How long was the visit?
   8. If YES: During the visit, did someone [doctor, nurse, pharmacist] go over [name of person with COPD]’s medications?
      1. If YES: Did they review the medications with you and the patient?
      2. If YES: Did they you or [name of person with COPD] about any problems with the medications?
      3. If YES: Were you given instructions/taught about their medications?
         1. If YES: were you able to understand what you were told or taught?
         2. If NO: Do you wish someone would have reviewed them with you as a caregiver?
      4. If YES: Were changes made to [name of person with COPD] medication list?
         1. If YES: Were the reasons for the changes explained?
      5. If NO: Did you wish they gave you information about the patient’s medications from a caregiver’s perspective?
         1. If YES: What would you have wanted to be taught or told?
   9. If YES: Did someone show you how to help [name of person with COPD] use their inhaler(s) or did you receive any other information about the patient’s COPD?
      1. What was told or taught to you?
      2. Were you able to understand this information and/or teaching?
         1. If NO: What was hard to understand?
      3. If NO: Did you wish they provided more information and teaching about COPD and inhaler use in general?
   10. If YES (reference 4.2): Were you told or taught something different during the clinic visit compared to when [name of person with COPD] was in the hospital?
       1. If YES: What was different?
   11. If YES: Were you told or taught about the new things to take care of [name of person with COPD]’s COPD?
       1. If YES: What were they?
5. Did the [doctor/nurse/pharmacist] and [name of person with COPD] decide on anything new, like a new medication or a change in the dose to take care of [name of person with COPD]’s COPD?
   1. If YES: Were you told or taught about the new things to take care of [name of person with COPD]’s COPD?
   2. If NO: What prevented you from coming to the visits?
   3. If NO: Would a tele-visit have been easier for you or the patient to attend the visit?
      1. If YES: Why would it have been easier?
      2. If NO: Why would it not be easier?
6. Did you or [name of person with COPD] schedule any other clinic visits with the doctor/nurse/pharmacist or with someone else?
7. Note to the interviewer: this and the following are open-ended follow up questions
8. On a scale of 1-5, how prepared did you feel to take care of [name of person with COPD] your care recipient was after the clinic visit? Likert scale: 1= Very prepared; 2=Prepared; 3=Neither prepared or not prepared; 4=Not prepared; 5= Not prepared at all
9. What challenges did you face during the clinic visit, if any?
10. Was any information missing that you needed for you to care of [name of person with COPD]?
    1. If YES: What was missing?
11. Were there any errors/mistakes in the education or information provided to you?
    1. If YES: What mistakes did you notice?
12. What impact did this have on taking care of [name of person with COPD]’s COPD?
13. Did you have any questions for the doctor/nurse/pharmacist that were not answered?
    1. If YES: what were they?
14. What things about the clinic visit worked well?
15. Were you able to use the education/information you got when caring for after the clinic visit?
16. Is there anything that could have been done differently during the clinic visit to better help you take better care of [name of person with COPD]?

**Intervention Technology skills/needs/comfort**

*We hope to design a clinic visit that can happen over video post-discharge to help with taking your medications for COPD.*

1. Have you ever helped [name of person with COPD] with a telehealth visit with their doctor or nurse?
   1. If YES: Can you tell me about [name of person with COPD]’s telehealth visit?
   2. If YES: What worked well during the visit?
   3. If YES: What didn’t work well?
   4. If YES: Do you think that anything was missed compared to an in-person clinic visit?
      1. If YES: What was missed?
   5. If NO: What has kept the patient from having a video visit?
   6. Is there anything that we can do to make it easier?
2. Would you be willing or able to help [name of person with COPD] with a telehealth visit?
3. If you had to share any lessons learned, tips, and information (that you had previously wished for yourself) to help caregivers of patients with COPD, what would you include?

**S5. TELE-TOC Phase 2 Organization Leader Interview Guide**

**Baseline Questions**

1. How would you describe your role and responsibilities as an organizational leader?
2. In what ways does your role interface with COPD patient care?
3. In what ways does your role interface with the delivery of tele-medicine?

**Implementation Considerations and Plan**

1. Who do you think should make-up the tele-visit clinical team? ​
   1. PCP? Pulmonologist? Nurses? Pharmacists?​
2. Are there any types of providers who should not be used to provide these visits?​
3. What type of support do you think will be needed by type of clinical team?​
4. What kind of training do you feel should be required (for patients and for tele-visit team?)​
5. What materials do you think will be needed for a successful visit?​
   1. For ensuring patients’ self-management?​
   2. For successful medication reconciliation?
6. What suggestions do you have to optimize communication (and documentation) between the inpatient and tele-visit teams? Between the tele-visit and the outpatient teams?  ​
7. Do you think that we need to create a tele-visit template note for the program? ​
   1. If so, what do you think needs to be included in the template note? ​
   2. And who should be able to access it? ​
   3. And where should it stored in the EHR? ​
8. Do you have any concerns about this type of intervention?​
   1. How can these concerns be mitigated/prevented?​
9. How can our tele-visit best reach UChicago Medicine's patient population?​
10. Please expand on strategies to best support patients with low health literacy.​
11. Please expand on strategies to best support patients with limited technology access/tech literacy.

**General Tele-visit Navigation**

1. To your knowledge, please describe the current workflow used for clinicians to access/conduct tele-visits.​
2. What is the current standard platform used for tele-visits?​
   1. What works well about this platform? ​
   2. What could be improved?​
   3. Are there alternative platforms currently in use?​
3. What tools/steps are necessary to ensure thorough medication reconciliation can be performed via tele-visit?​
   1. Are there any metrics to measure this?​
4. What tools/steps are necessary to ensure thorough inhaler education can be performed via tele-visits?​
   1. Are there any metrics to measure this?​
5. What are tools that help streamline the process of tele-visit navigation?
6. Who typically works with patients to get their tele-visits scheduled?​
   1. Does this differ in the inpatient vs. outpatient setting?​
7. What is possible, given the current infrastructure, for appointment reminders?​
   1. Can the patient portal be utilized?​
   2. Can email be utilized?​
   3. Can texting be utilized?​
   4. Would a text-only/messaging-based visit option be achievable?​
   5. What would be the limitations?

**Appointment Duration/Frequency**

1. How many visits would be sufficient for achieving medication management?​
   1. How would this differ for high-risk vs low-risk patients? ​
   2. How would this differ for high health literacy vs low health literacy patients?​
2. How long do you feel is an appropriate time for these visits to be scheduled?​
   1. 20 minutes/30 minutes/40 minutes?​
   2. How would this differ for high-risk vs low-risk patients? ​
   3. How would this differ for high health literacy vs low health literacy patients?​
3. What would be an appropriate frequency of these visits?​
4. What is the optimal timeframe for these tele-visits to occur?

**Final Thoughts**

1. What metrics (if any) are used to: ​
   1. Determine success of medication review during COPD outpatient visits?​
   2. Determine success of COPD education in outpatient visits?​
   3. Determine success of medication review during tele-health visits?​
   4. Determine success of COPD education during tele-health visits?​
   5. Evaluate the transition of care for previously hospitalized patients?​
2. To your knowledge, what is the overall patient experience/satisfaction for UChicago Medicine's current tele-health practices?​
   1. What can be improved?​
   2. What is working well?​
3. To your knowledge, what is the overall staff experience/satisfaction for UChicago Medicine's current tele-health practices?​
   1. What can be improved?​
   2. What is working well?​
4. Any additional thoughts on the intervention and its implementation?​
   1. Tips?​
   2. Concerns?

**S6. TELE-TOC Phase 2 Clinician Interview Guide**

**Baseline Questions**

1. Please describe your role and how it relates to patient care delivery. How many years have you been in practice?
2. How are you involved in patient care in clinical settings (inpatient, outpatient)? (Prompts: what are your clinical roles/responsibilities?)
3. Have you ever conducted a telemedicine visit? What is your overall experience with conducting tele-visits?
4. What is your experience with conducting tele-visits?
5. Do you have experience providing care to patients with COPD?
   1. If yes, how many years have you provided care for patients with COPD? About how many patients do you care for with COPD per week? Per month?
   2. If no, are you interested in providing care to patients with COPD?

**Tele-visit Intervention Description**

*Interviewer – use this time to describe the intervention to the clinician/clinicians using corresponding slides (slides 4-9).*

**Example Timelines for Discussion**

*Interviewer – use this time to walk clinicians through the timeline as outlined on slide 10.*

**Implementation Considerations & Plan**

1. Who do you think should be a part of a tele-visit clinical team?
2. What training/experience do you think clinicians of a tele-visit team need?
3. What type of tele-visit documentation would be required for this type of visit?
4. What type of technical support do you think a clinician would need?
   1. Would it be helpful to have someone to connect/troubleshoot with the patient before the clinician starts a telemedicine visit?
   2. Would it be helpful to have a separate computer and screen from the telemedicine screen for clinicians to document?
   3. Should there be a standardized tele-visit template?
      1. If yes – what should we capture in the note? Where should it be in EPIC? Who should have access to it?
   4. What other support/materials would be needed for a successful visit?
5. What kind of training would be required (for patients and for the tele-visit team?)
6. How could the tele-visit affect current inpatient to outpatient discharge planning?
7. How should the communications (and documentation) between inpatient to tele-visit teams occur?
8. How should the communications (and documentation) between the outpatient and tele-visit teams occur?
9. What are your main concerns about the tele-visit intervention?
   1. How can these be mitigated/prevented?
10. How can our tele-visit best reach UChicago Medicine’s patient population?
    1. Please expand on strategies to best support patients with low health literacy.
    2. Please expand on strategies to best support patients with limited technology access/tech literacy.

**General Tele-visit Navigation Questions**

Scheduling the visit -

1. If you are currently conducting telemedicine visits, what would you change to make the visit easier for you? For the patient?
2. What steps are needed to achieve an **ideal workflow** to schedule a tele-visit for an inpatient being discharged? **Who** should schedule a tele-visit?
3. Who should work with the patient to schedule?
   1. Should the patients' caregivers be involved in this process?
   2. Should there be a navigator/CHW?
   3. When would be the optimal time to start the scheduling process?
   4. How would this differ for high-risk vs low-risk patients?
   5. How would this differ for high health literacy vs low health literacy patients?
   6. How would this differ for patients with technology access vs without?
   7. How would this differ for patients in vs. out of network?
   8. How would this differ for patients discharged to home vs. secondary location?
4. What reminder method would be most effective to ensure patients’ attendance?
   1. Text
   2. Email
   3. Phone call
   4. Patient portal reminder
   5. Paper reminder
5. How would this differ for patients who are:
   1. High vs. low risk?
   2. High vs. Low health literacy?
   3. With vs. Without technology access?
   4. Discharged to home vs. Secondary location?
6. How many reminders do you think are needed?
7. When, in the transition of care process, should these appointment reminders be sent?

Accessing the visit -

1. Please describe an ideal workflow that can work for clinicians to access/conduct tele-visits. How comfortable are you seeing patients via tele-visit?
   1. Which team members are involved in tele-visit set-up?
   2. What would you change about this workflow to make the process easier for your team?
   3. What are tools that help streamline the process of tele-visit navigation?
2. How are tele-visits typically conducted?
   1. i.e - synchronously via phone, over video via Zoom, (OR) asynchronously
3. How long do you think a tele-visit should last?
4. How soon after discharge should the initial tele-visit be scheduled?
   1. 3 days
   2. 7 days
   3. Variable – selected by discharging MD
5. Which clinician do you think would be most appropriate for conducting the visit?
   1. Patient’s PCP
   2. Pulmonologist
   3. RT
   4. Nurse
   5. APN
   6. Team: (specify)
6. Do you think that the clinician(s) that you indicated above has the time/capacity for telemedicine visits?
   1. How would this differ for patients who are high vs low risk?
   2. How would this differ for patients with high vs low health literacy?
   3. How would this differ for patients with vs without technology access?
   4. How would this differ for patients discharged to home vs a secondary location?

Initial Visit/Follow-ups -

1. What information from the hospital stay would be helpful for the clinician conducting the tele-visit to have?
   1. When would be the most convenient time to receive this information?
   2. Who would best be suited for sending this information to the tele-visit team?
2. After the initial tele-visit, how many follow-up telemedicine visits do you think are needed to prevent further hospitalizations?
   1. How would this differ for patients who are high vs low risk?
   2. How would this differ for patients with high vs low health literacy?
   3. How would this differ for patients with vs without technology access?
   4. How would this differ for patients in network vs out of network?
   5. How would this differ for patients discharged to home vs a secondary location?
3. How would the subsequent visits differ from the initial visit?
4. If more than one tele-visit is recommended, please tell us how you think the timing of subsequent visits might change?
   1. How would this differ for patients who are high vs low risk?
   2. How would this differ for patients with high vs low health literacy?
   3. How would this differ for patients with vs without technology access?
   4. How would this differ for patients in network vs out of network?
   5. How would this differ for patients discharged to home vs a secondary location?
5. Should the subsequent visit be before and after the outpatient visit? What will the focus of these sessions be?

**Final Thoughts**

1. Any additional thoughts on the intervention and its implementation?
   1. Tips?
   2. Concerns?

**S7. TELE-TOC Phase 2 Patient Interview Guide**

**Baseline Questions**

1. On a scale of 1 to 5 (with 1 being not at all and 5 being very much), how confident are you with using the internet?
2. Have you ever used FaceTime, WhatsApp, Zoom, or other video chat technology for non-medical reasons?
   1. If yes –
      1. Which ones have you used?
      2. How would you rate your experience? Use a scale of 1 to 5 with 1 being not good and 5 being very good.
      3. How confident are you using these technologies?
3. Have you ever used FaceTime, WhatsApp, Zoom, or other video chat technology for a healthcare tele-visit before?
   1. If yes-
      1. Was it a good experience? Why/why not?
      2. Did you have any technical difficulties during the televisit?
      3. Who was the visit with?
      4. What type of device did you use? [A cellphone?, ipad? Computer?]
      5. Did you use the video?
   2. If no-
      1. Would you be interested in having tele-visits? Why/why not?
      2. Do you have a device at home that could be used to access a tele-visit?
      3. Would you need help using a device for a tele-visit?
4. What would encourage you to take part in a tele-visit from home (either video, phone, or through chat)?
5. What would keep you from taking part in a tele-visit from home (either video, phone, or through chat)?

**Appointment Set-Up**

1. When do you feel would be the ideal time to schedule a tele-visits?
   1. During your hospital stay vs after?
      - 1. If after – how soon after discharge?
   2. What time of day would work best for you? Why?
   3. What days of the week would work best for you? Why?
2. What instructions would you appreciate getting to set up a tele-visit?
   1. Would it be helpful to have someone show you how to set up a tele-visit?
   2. Would you prefer a physical appointment reminder (on paper), or a digital appointment reminder (telephone/email/text).
3. Have you ever used MYChart , the UChicago Medicine patient portal?
   1. If yes,
      - 1. What was easy? What was hard?
   2. If no, why not?
4. What platform, if not MyChart (patient portal) would be easiest to use for your tele-visit?
5. Would you be interested in
   1. A visit conducted by video?
   2. A visit conducted by a phone call?
   3. A visit conducted via text message?
   4. Please rank all these options from most preferred to least preferred and explain.

**Appointment Reminders**

1. How many reminders do you think you would need before your scheduled tele-visit?
   1. What would be helpful to include information in these reminders?
   2. If yes, what information would be helpful?
   3. When would be the best time for you to receive these reminders?
2. How would you prefer these reminders be delivered?
   1. Text? Email? Patient portal (MyChart)?

**Appointment Duration/Frequency**

1. How many tele-visit do you think you would you take part in? (examples of self-management visits include when a clinician checks in with you about your COPD management and/or education, or when a clinician teaches you how to use medication)
   1. Is three visits too many?
   2. Is one visit too few?
2. How long do you feel do you think a televisit should last?
   1. 20 minutes/30 minutes/40 minutes?
3. If the tele-visit program offers more than one visit, how much time should pass between each visit?

**Tele-visit Content**

1. Would you feel comfortable having a nurse have the tele-visit with you?
   1. Why or why not?
2. Would you feel comfortable having a pharmacist have the tele-visit with you?
   1. Why or why not?
3. What do you feel would be most useful topics to be covered during these visits?
   1. Would you be interested in going over your medication list? Why/why not?
   2. Would you be interested in receiving education about your COPD? Why/why not?
   3. Would you be interested in receiving instructions/teaching on how to use your inhalers? Why/why not?
   4. What questions would you want addressed during the visit?
4. Are there questions/concerns that you feel could not be addressed over the phone or on video?

**Implementation Considerations**

1. What would motivate you to participate in tele-visits?
2. Do you own a device that could be used for a tele-visit?
3. Do you own a device that you can use for MyChart?
4. On a scale of 1 to 5 (1 being not at all comfortable, 5 being very comfortable), how comfortable are you using the internet?
   1. Do you need help to use the internet?

**Final Thoughts**

1. Do you have any additional thoughts on using tele-visits?
   1. Tips for other patients?
   2. Tips for clinicians?
   3. Concerns?

**S8. TELE-TOC Phase 3 Clinician Interview Guide**

*To get started, I will have you login to MyChart like you are going to conduct a telehealth visit. While you do this, please verbalize your thoughts regarding issues you are encountering or any thoughts that you have while you navigate to MyChart.*

*Now, we would like to talk about you experience with video visits you have conducted before.*

1. Have you conducted a visit like this before to help patients manage their COPD post discharge (video visit for COPD self-management)?
   1. If yes- in what setting, tell us more
   2. Was it virtual or in person? Where did this visit happen?
2. How did this mock COPD visit compare to other virtual visits you have conducted in the past?
3. How did it compare to other in-person visits you conducted had in the past?

*Now, we would like to move on to discuss what you thought about the workflow of the video visit.*

1. What, if anything, would you change about the mock COPD medication visit?
   1. The order of topics discussed?
   2. Teaching inhaler technique over video?
   3. When med rec occurs?
   4. Scheduling visits for patients?
2. How did you feel your interactions were during the mock video visit? Did you feel the patient was engaged more/less than an in-person visit?
3. How would you rate on a scale from 1 (did not understand at all) to 5 (understood completely) your patient’s understanding of their self-management practices?
   1. Why did you give this rating?
4. On a scale from 1 (very difficult) to 5 (very easy), how easy or difficult was it for you to
   1. Schedule the visit? -
   2. Navigate to the visit?
   3. Use videoconferencing for the visit
   4. Use/follow the visit note
   5. Log onto the visit?
   6. Log off of the visit?
5. What part of the mock visit, if anything, was difficult or confusing?
   1. Was it difficult/confusing to: (probe for why)
      1. Figure out how to log on?
      2. Turn your camera on/unmute yourself?
      3. See or hear your patient?
      4. Scheduling the next video visit
      5. Teaching inhaler technique to patients
      6. Conducting med rec.
      7. Using the visit note as a guide
      8. Inputting information into the visit note
6. Did you think the topics discussed in the visit followed a logical workflow?
7. Would you change anything about the workflow of the visit in general?
8. Do you think you would benefit from assistance for navigating to/completing the mock visit? What specific parts require assistance?
9. Did you find the visit note to capture everything you needed for the visit?
   1. Why or why not?
10. How did using the note feel to use compared to other notes you have used previously?
11. How did you feel using videoconferencing to complete this visit?
    1. Was there anything about this that made it easier or more difficult to conduct the visit?.
12. On a scale of 1-5 how much do you agree with the following statement:
    1. I found the workflow unnecessarily complex
    2. I thought the workflow was easy to navigate
    3. I think that I would need the support of a technical person to be able to successfully conduct a COPD video visit
    4. I thought the COPD video visit was easy to access
    5. I would imagine that most patients would be able to access COPD video visits easily
    6. I found the COPD video visit cumbersome
    7. I felt confident conducting the COPD video visit
    8. I needed to learn a lot of things before I could conduct the COPD video visit
13. On a scale of 1-5, 1 being very poor and 5 being great, how would you rate the experience of this mock visit?
    1. 1: Very poor
    2. 2: Poor
    3. 3: Average
    4. 4: Good
    5. 5: Great

**S9. TELE-TOC Phase 3 Patient Interview Guide**

**TELE-TOC Visit Workflow**

*For these next couple of questions we want to see if you have had any prior experience with this type of visit.*

1. Have you had a visit like this (i.e., video visit for COPD medication management) after you were discharged home:
   1. Prompts:
      1. Any visit after you left the hospital? When did this occur? Where did it occur (clinic, home)
      2. Any medication management visits ever that were done with video visits?
         1. Did they check which medications you should be taking?
         2. Did they teach you how to use your inhalers?
2. How did the mock COPD medication visit compare to other virtual (or in-person) visits you have had in the past?
   1. Prompts:
      1. How did it compare going over your medications? (i.e., which ones to use and when)
      2. How did it compare with other teaching about inhaler use ? (i.e., what the steps are for getting the inhaler medications in to the lungs)
      3. Any other thoughts about this mock copd medication visit

*For the next few questions, we now want to ask you about this mock visit we just had.*

1. How did you feel about the mock COPD medication video visit? (note to interviewer: use the process workflow map for additional prompts and follow-up any prompts from the think alouds)
   1. Prompts:
      1. Being in your home
      2. Visit reminders
      3. Logging into the visit using EPIC/MyChart
      4. Going over meds/inhaler technique
      5. Talking to your clinician on the computer
      6. Using a device for this visit
2. What part of the mock visit, if anything, a positive experience
   1. Was it helpful: (probe for why)
      1. To be able to be at home and not have to come into clinic in person?
      2. Go over your medications –which ones to take and when
      3. Being taught how to use your inhalers(s)?
      4. Anything else?
3. What part of the mock visit, if anything, was difficult?
   1. Was it difficult/confusing to: (probe for why)
      1. Figuring out how to log on?
      2. Turn your camera on/unmute yourself?
      3. Seeing or hearing your clinician?
      4. Something else?
4. What did you think about the order in which your clinician discussed your medications, inhalers, and other topics related to your COPD? Was it easy to follow? Any suggestions?
5. What, if anything, would you change about the mock COPD visit?
   1. For example:
      1. When inhaler education was discussed
      2. When medication discussions occurred
      3. When they discussed previous hospital/ER visits
      4. Answering your questions
      5. Getting your next appointment schedule
6. We know that technology doesn't always work as planned.  Would you have wanted help/would want help joining this visit in the future?

(if yes) prompt a or b

- 1. Is there someone you can ask to assist you at home with TELE-TOC?
  2. Would you need someone from our team to help guide you through the visit?

**TELE-TOC Visit Content**

*Now that we have discussed what you liked about the visit, we know would like to learn more about the topics we covered:*

1. Did you think the information we provided was helpful to you to manage your

COPD (If yes): How was it helpful?

- 1. For:
     1. Improving awareness of your COPD condition
     2. Building your self-management skills
        1. How to use your inhalers
        2. How to use your other medications/O2 equipment
     3. Fixing any discrepancies in your current medications with our med review
     4. Answering your questions
     5. Keeping you out of the hospital/ER
  2. If no: What would have made this visit more helpful?

1. How easy did you find it to follow along the information and instructions provided by the clinician during the visit? (e.g. inhaler education) What do you think contributed to this?
2. Did the visit with the clinician help build your confidence in taking care of your COPD at home? How so?
3. Did the visit with the clinician help with your understanding of how to use the inhaler and other medications at home to manage exacerbations? How?
4. What, if anything, would you suggest we add to the mock visit?
5. What, if anything, would you suggest we remove from the TELE-TOC visit?
6. On a scale from 1 (very difficult) to 5 (very easy), how easy or difficult was it for you to:
   1. Schedule the visit?
   2. Navigate to the visit?
   3. Log onto the visit?
   4. Log off of the visit?
   5. Turn your camera on?
   6. Unmute yourself?
   7. Hear your clinician?
   8. Using your device for the visit?
   9. [if any other items from above raised as issue- f/u here with the scale)
7. On a scale of 1-5 (1 - Strongly disagree, 5 – Strongly agree) how much do you agree with the following statement:
   1. I would like to have a COPD video visit after each hospitalization for COPD
   2. The COPD video visit too hard
   3. The COPD video visit was easy to do
   4. I need someone to help me with the COPD video visit
   5. I think the COPD video visits would be easy for most people
   6. The COPD video visit was too long
   7. I felt confident joining the COPD video visit
   8. I needed to learn a lot of things before I could access the COPD video visit.
8. On a scale of 1-5 (1 - Strongly disagree, 5 – Strongly agree) how much do you agree with the following statement:
   1. It was easy to do this video visit
   2. I understood what my clinician told me about my COPD care/medications
   3. It was easy to follow what my clinician told me
9. On a scale of 1-5, 1 being very poor and 5 being great, how would you rate the experience of this mock COPD video medication visit?
   1. 1: Very poor
   2. 2: Poor
   3. 3: Average
   4. 4: Good
   5. 5: Great
10. On a scale of 1-5, 1 being not at all, 5 being extremely helpful, how helpful did you find the mock visit for the following:
    1. Improving how you use your inhaler(s)
    2. Knowing when to use your medications
    3. Knowing what medications to use
    4. Improving your confidence to manage your breathing
    5. Improving your confidence of not needing to go to the hospital
11. Is there anything else you wanted to tell us that could make this visit better?
